# High Throughput Identification of Antihypertensive Peptides from Fish Proteome Datasets

**DOI:** 10.3390/md16100365

**Published:** 2018-10-02

**Authors:** Yunhai Yi, Yunyun Lv, Lijun Zhang, Jian Yang, Qiong Shi

**Affiliations:** 1 School of Applied Chemistry and Biotechnology, Shenzhen Polytechnic, Shenzhen 518055, China; yiyunhai@genomics.cn (Y.Y.); c7zlj@szpt.edu.cn (L.Z.); 2 BGI Education Center, University of Chinese Academy of Sciences, Shenzhen 518083, China; lvyunyun@genomics.cn; 3 Shenzhen Key Lab of Marine Genomics, Guangdong Provincial Key Lab of Molecular Breeding in Marine Economic Animals, BGI Academy of Marine Sciences, BGI Marine, BGI, Shenzhen 518083, China

**Keywords:** antihypertensive peptide, fish protein hydrolysate, high throughput identification, hypertension, collagen

## Abstract

Antihypertensive peptides (AHTPs) are a group of small peptides with the main role to block key enzymes or receptors in the angiotensin genesis pathway. A great number of AHTPs have been isolated or digested from natural food resources; however, comprehensive studies on comparisons of AHTPs in various species from the perspective of big data are rare. Here, we established a simplified local AHTP database, and performed in situ mapping for high throughput identification of AHTPs with high antihypertensive activity from high-quality whole proteome datasets of 18 fish species. In the 35 identified AHTPs with reported high activity, we observed that Gly-Leu-Pro, Leu-Pro-Gly, and Val-Ser-Val are the major components of fish proteins, and AHTP hit numbers in various species demonstrated a similar distributing pattern. Interestingly, Atlantic salmon (*Salmo salar*) is in possession of far more abundant AHTPs compared with other fish species. In addition, collagen subunit protein is the largest group with more matching AHTPs. Further exploration of two collagen subunits (col4a5 and col8a1) in more fish species suggested that the hit pattern of these conserved proteins among teleost is almost the same, and their phylogeny is consistent with the evolution of these fish species. In summary, our present study provides basic information for the relationship of AHTPs with fish proteins, which sheds light on rapid discovery of marine drugs or food additives from fish protein hydrolysates to alleviate hypertension.

## 1. Introduction

The prevalence of cardiovascular diseases is mainly caused by high blood pressure [1,2]. Clinical trials have observed that lowering systolic blood pressure would reduce cardiovascular morbidity and mortality significantly [3]. However, serious side effects, such as kidney function deterioration and electrolyte abnormalities, occurred with utilization of various antihypertensive medication [3,4]. Lisinopril, captopril, losartan, azilsartan, atenolol and hydralazine are the common drugs for hypertension treatment [3]. Among them, angiotensin converting enzyme (ACE) inhibitors are preferred antihypertensive drugs [5], as ACE is responsible for the conversion of angiotensin I (Ang I) into vasoconstrictor angiotensin II (Ang II) and the inactivation of vasodilator bradykinin [6,7]. Given the adverse effects of synthetic drugs, there is a growing demand for antihypertensive agents from natural food resources over the past decades since the discovery of natural antihypertensive peptides (AHTPs) from a snake venom [8,9].

AHTPs, a group of small bioactive peptides affecting the workflow of renin-angiotensin system (RAS), commonly are enzyme inhibitors (such as renin and ACE inhibitors) and angiotensin receptor antagonists [6,7]. The most effective and popularly studied molecule is ACE inhibitory peptide [10]. Food-derived AHTPs have been isolated and further validated in vitro and in vivo from various repositories of plants and animals, including milk, soybean, chicken and fish protein hydrolysates [11,12,13]. Commercial products from milk and fish hydrolysates have been popular in markets [14,15]. VPP and IPP are well-known ACE inhibitory lactotripeptides for functional foods, although their effects cannot be comparable to drugs while no adverse effects happened [12].

These natural food proteins provide thousands of templates to develop antihypertensive drugs and functional food supplements [16,17]. The public Database of Antihypertensive Peptides (AHTPDB, http://crdd.osdd.net/raghava/ahtpdb/) had collected large amounts of AHTPs by 2015 for better exploitation of antihypertensive agents [18]. Among these, fish hydrolysates have aroused enormous interests for their inexhaustible feature and high nutritional value [19,20]. Bioactive peptides with antimicrobial, antioxidant, anti-obesity, antihypertensive and cryoprotective effects from fish proteins in muscle, skin and even waste products have been reported [19,21,22,23]. Characterization of AHTPs in fish protein hydrolysates have been conducted in various species, such as salmon, tuna, grass carp and dace [24,25,26,27].

Protease hydrolysate components of fish, especially from fish waste products, have been studied for ages. Related engineering reports dedicating to purification techniques, enzymes, crude materials and efficiency assessment of mature products are increasing rapidly [28]. However, the overall knowledge about fish protein content from perspective of big data, and what sort of proteins are potential candidates with the most abundant AHTPs, have not been well examined yet. To figure these out, we performed comparative analyses in this present study for high throughput identification of AHTPs by in situ mapping of whole proteomes from 18 fish species, ranging from ancient coelacanth, spotted gar to teleost fish in Perciformes. We found that Atlantic salmon has more AHTP hits than other fish species. We also identified that collagen subunit protein is in possession of the most plentiful AHTPs. Anyway, our current work initiated a comprehensive investigation into potent AHTPs from protein content in various fishes using big data, which will present instructions for alternatives of raw materials in production of AHTPs.

## 2. Results

### 2.1. Construction of a Local AHTP Database

**Figure 1:**
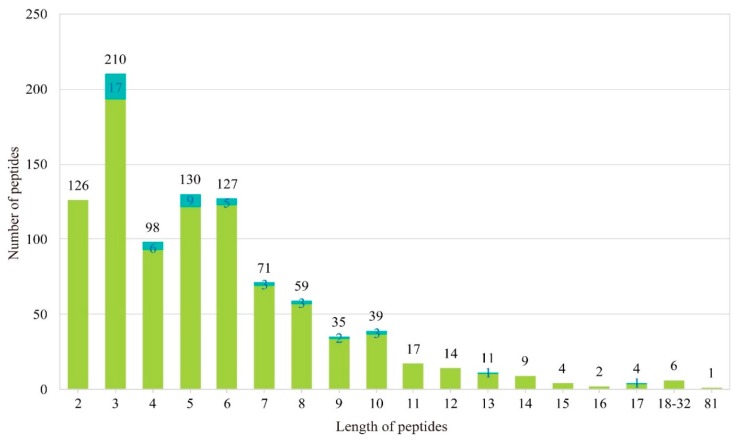
Overview of peptide length vs. numbers in our local AHTP database. Blue boxes within some groups denote the numbers of peptides that we generated in this study, which potentially have high activities for antihypertension.

We established a local AHTP database (Table S1) with 963 records, which was created manually from the public AHTPDB and previously published reports. We employed the same unique AHTPDB ID, except for 42 entries (larger than 6979 in ID number) that we appended. Detailed information of sequence, length, molecular weight, half maximal inhibitory concentration (IC_50_), isolating source, animal model for in vivo test, purification method, IC_50_ determination assay, isoelectric point (PI) and decrease in systolic blood pressure is summarized in Table S1. Interestingly, nearly 20% AHTPs are tripeptides, and 85% AHTPs are between two and eight amino acids (aa) in length (Figure 1). IC_50_ ranges from 0.01 to 12,000 µM, while half of them are less than 50 µM. Among them, 148 peptides are with in vivo tests on animal model of spontaneously hypertensive rats, in which 118 entries generated decrease in systolic blood pressure.

### 2.2. Identification of AHTPs with High Activity

To identify AHTPs more explicitly, we picked 50 AHTPs with the highest activity (see more details in Table S1) to search against the protein datasets of 18 fish species (find corresponding names in Table 1 and Table S2), including coelacanth (*Latimeria chalumnae*) and 17 teleost species. The species selection obviously tends to elucidate AHTPs down the evolutionary timeline. The specific alignment results were provided in Table S3, with a clear exhibition of the locations for each matched AHTP on its corresponding protein.

**Figure 2:**
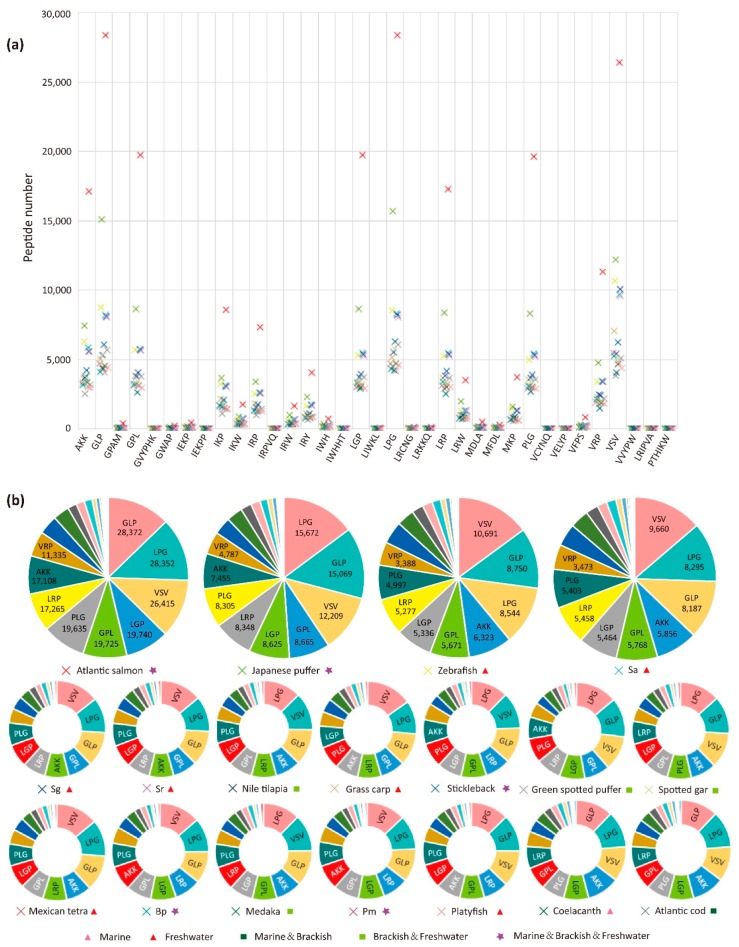
Statistics of the mapped AHTPs. (**a**) AHTP hit number in each species. Crosses with the same color represent the same species. (**b**) Composition of the mapped AHTPs in each protein dataset from the 18 examined fish species. Arrangement of the pie charts is based on the total hit number (from the greatest to the least) in one species. Abbreviations of fish species: Sa, *Sinocyclocheilus anshuiensis*; Sg, *S. grahami*; Sr, *S. rhinocerous*; Bp, *Boleophthalmus pectinirostris* (Blue-spotted mudskipper); Pm, *Periophthalmus magnuspinnatus* (Giant-fin mudskipper).

However, 15 AHTPs did not match any protein, and among them 13 peptides are longer than 6 aa and 2 peptides are hexapeptides. In the 35 mapped AHTPs (Figure 2a), most tripeptides (such as GLP, LGP, LPG and VSV) have numerous hits in the 18 examined fish species (Figure 2b). Tetrapeptide, pentapeptide and hexapeptide were much less likely to match a protein. Interestingly, it seems that the hit number was also influenced by the aa composition of AHTPs. For example, the difference among IRP (Ile-Arg-Pro), IRY (Ile-Arg-Tyr) and IRW (Ile-Arg-Trp) is the last C-terminal aa; however, their hit numbers varied greatly (Figure 2a and Table S4). 

Values of Pearson correlation coefficient (Pearson’s r; relationship between total protein number and mapped protein number) of AHTP hit numbers in each type (Table S4) between spotted gar (*Lepisosteus oculatus*) and other 17 fish species, as well as between caved Mexican tetra (*Astyanax mexicanus*) and other 17 fish species, were perfectly correlated (r > 0.98). That is to say, when the hit number of an AHTP in one species is greater than another species, the hit number of other AHTPs would also be greater in general. The variance of each mapped AHTP and correlations of AHTP hit number between fishes suggest that different types of AHTPs have variable tendency to occur in organisms, while a similar AHTP hit pattern was conserved among the 18 examined fish species.

**Figure 3:**
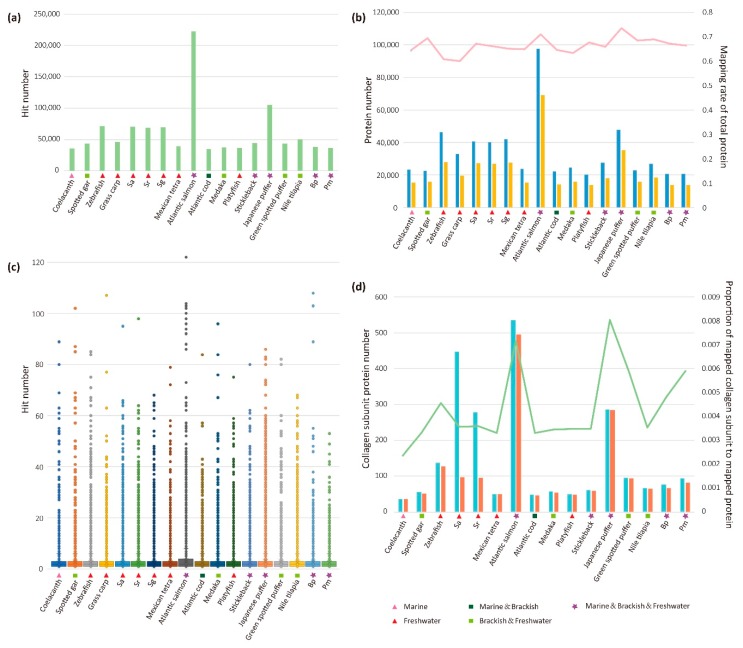
Statistics of AHTP hit numbers and mapped proteins. (**a**) Total AHTP hit numbers from the 18 examined fish species. (**b**) Summary of the total and mapped protein numbers, and corresponding mapping rates (the pink line). Blue boxes denote the total proteins while yellow boxes indicate the mapped proteins. (**c**) Statistics of AHTP hit number of each protein in the 18 fish species. Box plots indicate the median (bold line), the 25th and 75th percentages (box edges), the range (whiskers), and the outliers (dots). (**d**) Summary of the numbers for total and mapped collagen subunit proteins, and their ratio in mapped proteins (the green line). Light blue boxes denote the total collagen subunit proteins while orange boxes indicate the mapped collagen subunit proteins. The order was arranged in accordance with the phylogenetic relationship of the 18 examined fish species.

In all datasets, Atlantic cod (*Gadus morhua*) has fewest AHTP hits while Atlantic salmon (*Salmo salar*) is the most abundant in every type of AHTPs (Figure 2 and Figure 3a). Interestingly, the Top 11 AHTP categories (marked in Figure 2b) are the same in all 18 fish species. All these massive peptides scattered around a considerable amount of proteins in each species, with a mapping rate ranging from 0.59 to 0.74 (Figure 3b). Pearson’s r values of the 18 fish species indicated that the more total protein a species possesses, the more protein would be mapped (r = 0.995). It was the same between total protein number and total hit number (r = 0.981) in all species. Average AHTP hit number in each protein of the 18 fish species ranges from 2.3 to 3.3 (Figure 3c; with more details in Table S4). However, there are hundreds of outliers beyond the mean level (Table S5), accounting for almost a third of the mapped proteins. 

Overall, the potential AHTPs produced by proteins of Atlantic salmon would be more abundant than any other fish, suggesting its advantage as a healthy food. In addition, based on the classification of water ecotypes for residence of the 18 fish species, we found that it was not related to the total amount of matched AHTPs (Figure 3).

### 2.3. Analysis of Mapped Proteins

**Figure 4:**
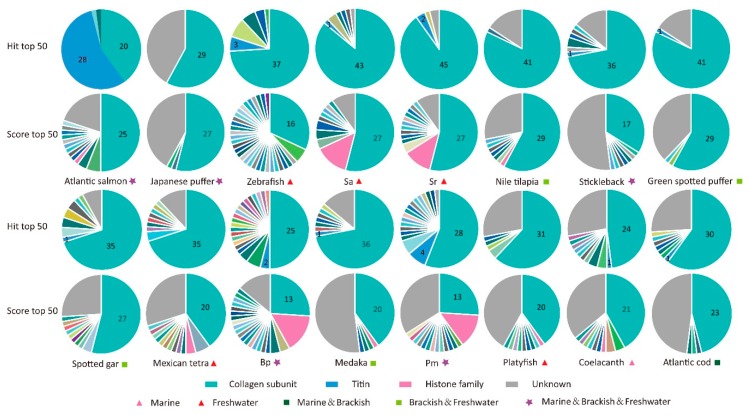
Statistics of protein category for the Top 50 hit and the Top 50 score in 16 fish species.

Since the AHTP hit number of every protein in each fish species (Figure 3c and Figure S5) supported a variety of matched AHTPs for the numerous mapped proteins, we summarized the categories of 50 proteins with top hits and top scores for reference (Figure 4 and Figure S6). As a large amount of sequences are unannotated in the protein datasets of golden-line babel fish Sg (*Sinocyclocheilus grahami*) and grass carp (*Ctenopharyngodon idella*), statistics was subsequently applied to the 16 other fish species. Surprisingly, among the 50 proteins with the most hits, collagen subunit proteins accounted for the largest part in general (Figure 4). Another protein, titin, prevailing in most species especially in Atlantic salmon, is the largest giant protein known so far. Taking the length discrepancy into account, categories of the Top 50 proteins with the highest scoring values were more diverse. The proportion of collagen subunit proteins descended in most species, and titin disappeared while proteins of histone family were identified in some fishes. It seems that titin has a great deal with matched AHTPs mainly due to its long length.

Categories of proteins with the first top hit and top score from the 18 examined fish species are listed in Table 1. Collagen type IV alpha 5 (col4a5) subunit and collagen type VIII alpha 1 (col8a1) subunit were commonly identified in most species. Meanwhile, in proteins ranked in the Top 50 of score values (Table S6), type IV, VIII and IX collagen subunits were the most extensive types. It is notable that none are fibril forming collagen. However, ancient coelacanth has a limited number of collagen subunit proteins (Figure 3d). Teleost fishes seem to evolve more members of collagen gene family (such as *col25a1*, *col8a1* and *col9a1*) and generate various transcripts, especially in the tetraploid Atlantic salmon and Atlantic cod. Similar observation exists in the tetraploid *Sinocyclocheilus* fishes (Sg, Sr and Sa) and Tetraodontiformes. These splice transcripts and isoforms (Figure 3d and Figure 4) would contribute to the generation of tremendous AHTPs under the hydrolysis with various hydrolases.

### 2.4. Deep Investigations into Two Collagen Subunits

**Figure 5:**
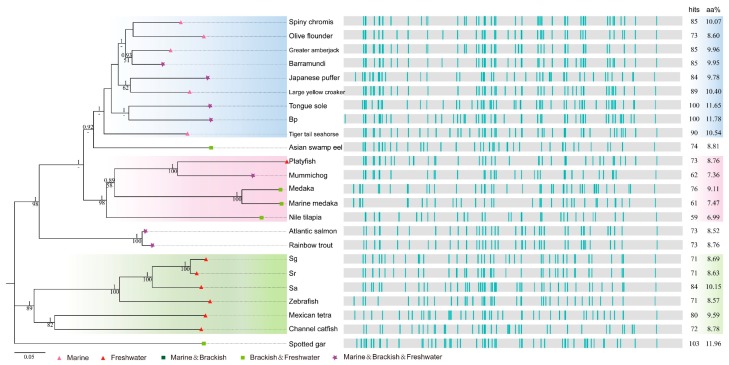
Phylogenetic analysis and AHTP localization of col4a5 in 24 fish species. Corresponding accession number of each protein sequence can be found in Table S7 and detailed AHTP localizations in Table S8.

**Figure 6:**
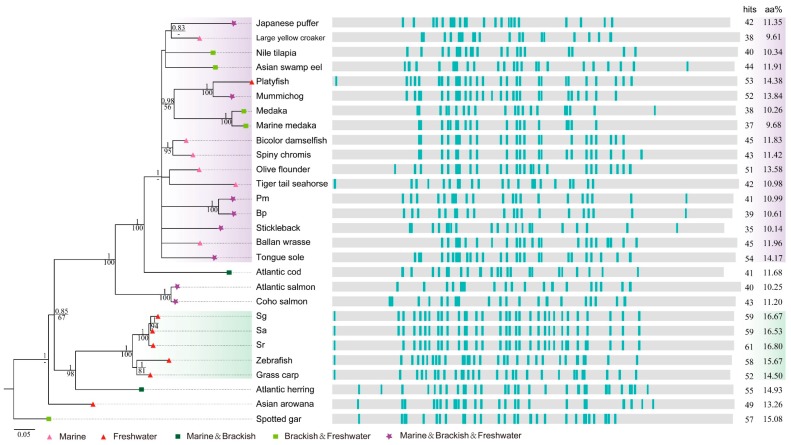
Phylogenetic analysis and AHTPs localization of col8a1 in 28 fish species. Corresponding accession number of each protein sequence can be found in Table S7 and detailed AHTP localizations in Table S9.

To learn more about the conserved collagen proteins, we collected homologous sequences of col4a5 and col8a1 (approximately 1685 and 740 aa, respectively) from more fish species (see more details in Tables S7–S9) for deep analysis. They are both structural component of basement membranes in extracellular matrix [29,30]. Phylogenetic analysis and localization of AHTPs in these two macromolecules were realized (Figure 5 and Figure 6). Interestingly, the phylogenetic analysis revealed that the evolutionary relationship of col4a5 and col8a1 coincided with the phylogeny topology of the selected fish species, when spotted gar was used as the outgroup (left in Figure 5 and Figure 6). The tree topology showed that if there is a closer relationship between certain two fish species, their sequences would have a more similar AHTP mapping pattern (right in Figure 5 and Figure 6). 

There are multiple GLPG repeating motifs in the collagen sequences, resulting in constant occurrence of triple AHTPs of GLP and LPG (Tables S8 and S9 and Figure 5 and Figure 6). The hits in C-terminal of col4a5 sequences, showing collinear alignment among 24 species in the positions around 1550-1560 (right of Figure 5), are IKP except one hit for IRP in Nile tilapia (*Oreochromis niloticus*). It was also revealed that col4a5 is more conserved than col8a1, as the latter has collinear alignment only among closely related species (right of Figure 6). In addition, all matched AHTPs in the col8a1 sequences are composed of three amino acids of G, L and P.

In the phylogeny of col4a5 (left of Figure 5), four major groups were obviously distinguished. Mainly clustered by genetic distance, the examined species included six fish species in Ostariophysi, salmonids in Protacanthopterygii, Beloniformes and Cyprinodontiformes in Acanthopterygii, as well as other fishes in Acanthopterygii (Table S8). However, in the phylogeny of col8a1, members in Acanthopterygii were converged into a larger group (Table S9). Atlantic cod, the species in Paracanthopterygii for separating Acanthopterygii from Protacanthopterygii perfectly, was a sister group to them. We then observed three major groups (left of Figure 6). 

Interestingly, the branch lengths of platyfish (*Xiphophorus maculatus*) and medaka (*Oryzias latipes*, *Oryzias melastigma*) in col4a5 are longer than those of other teleost, while salmonids (Atlantic salmon, Rainbow trout: *Oncorhynchus mykiss*) have shorter branch lengths (Figure 5), suggesting their protein evolutionary rates are distinguished for some reasons. Meanwhile, it seems that there is no correlation between the aa proportion of matched AHTPs and total hit numbers in col4a5 and col8a1 among different water ecotypes (Figure 5 and Figure 6) across the total examined 33 fish species (Table S7), suggesting a more complex collagen evolutionary pattern in the numerous fish species. 

## 3. Discussion

### 3.1. High-Throughput Identification of AHTPs

In this study, we identified myriad proteins (accounting for nearly 60% of each whole proteome from the examined 18 fish species) matching 50 AHTPs with the highest antihypertensive activity. In this case, with more AHTPs utilized, we would be able to map more proteins. In reverse, the more proteins a species possesses, the more AHTPs could be matched as indicated by our results.

If all AHTPs were used to identify potential peptides for antihypertension in fish proteins, it would be tremendous amount of mapping results; hence, we had to choose Top 50 AHTPs to conduct a representative analysis. This approach relies heavily on previously reported active AHTPs, thus the quality of established AHTP database is crucial for identification of novel bioactive AHTPs [31]. To facilitate data reutilization [13], we recommend unification of the concentration unit for enzyme inhibition assays, and establishment of a standard procedure to construct an updated real-time database.

In a number of studies, BIOPEP database [32] has been regarded as a comprehensive tool to identify peptides with various activities. Salmon col8a1 protein displayed different activities (as shown in Table S10), which were calculated by this in silico analysis. Interestingly, 118 peptides with ACE inhibitor activity were scored by PeptideRanker [33], but the majority are dipeptides and some peptides employed by our present study were in the front rank (Table S10). However, it is unclear whether they could be produced under physiological conditions. ExPASy PeptideCutter was employed to simulate digestive reactions of col8a1 by pepsin and trypsin, with 214 cleavages sites generated from all possible fragments (Table S10). These data indicate that the combination of various enzymes increases the number of peptides, although not all predicted AHTPs could be produced or some peptides occurred with unknown activities [34,35]. For example, AHTPs of GLP, GPL and LPG are always split by pepsin, while PGF, GDK, PGK and PQY, whose activities have not been proved yet, appeared as potential candidates according to the prediction of AHTpin [36] (http://crdd.osdd.net/raghava/ahtpin/). In fact, many platforms could identify AHTPs in protein hydrolysates in silico, demonstrating the advantages of bioinformatics for instructing us to perform subsequent physiological tests [34,37]. The actual situation, nonetheless, has numerous complex tasks to complete. The exploitation of novel enzymes is one of the keys to successful releasing of AHTPs from proteins. Except for pepsin, trypsin, alcalase, flavourzyme, neutrase and protamex, other novel proteinases are also waiting for exploration [38] to generate bioactive peptides more efficiently and productively.

Meanwhile, antihypertensive activity of many peptides in vivo is largely unknown. Among our 35 mapped AHTPs, only seven peptides were tested in vivo (Table S1): IWH, IKW, MKP, IWHHT, LIWKL, VELYP and IKP. Generally speaking, they are not rich in most species except the IKP. The top hit peptides usually have no data from in vivo test yet. It is worth investigating the relationship between their antihypertensive activity and their amount from a whole proteome view. Therefore, further bioactive validation is indispensable to the understanding of AHTPs. What is more, the composition of peptides is a crucial factor to affect the activity of AHTPs. Composition analysis of AHTPs have revealed that Gly (G) and Pro (P) are prevalent in dipeptides and tripeptides [36]. Quantitative structural activity relationship (QSAR) is a powerful tool to predict their activity [39]. Molecular docking and other prediction model are also dedicated to find novel AHTPs and further provide guidelines to subsequent preliminary tests [37,40]. It was also reported that peptides rich in Arg (R), Val (V) and Leu (L) with lower molecular weight exhibited a higher antihypertensive activity [41]. Based on these characteristics, we should make a restricted choice to select crude materials for AHTPs. Thereby, related exploration into functional supplements and drugs originated from fish still have a long way to go.

### 3.2. Potential Utility of Collagens

Compared with other proteins, the collagen family has a distinct characteristic of triple-helical Gly-X-Y structure [30]. This gives rise to the repeat occurrence of AHTPs of GLP and LPG. Furthermore, their bioactivities demonstrate their remarkable advantages in various fields [42,43]. For example, Vastatin, an endogenous antiangiogenic polypeptide from non-collagenous C-terminal globular domain of col8a1, has been reported to be promising in fighting against glioblastoma [44]. Elevated expression of type VIII collagen in the serum of patients with various cancers suggested its relationship with angiogenesis [45]. In addition, type IX collagen was required for the formation of vascular plexus [46], and the enzymatic fragments of salmon col7a1 isoform X5 also showed ACE inhibitory activity [37]. Previous studies also reported that collagen subunits contain multiple host defense peptides [47]. What is more, some collagen-derived peptides have antioxidant and anti-inflammatory capacities [48], and even can suppress allergic responses [49]. In this present study, multiple collagen subunits aroused our attention since their mapping ratios are much higher compared with other proteins. Titin, troponin, actin and myosin are frequently employed for generation of AHTPs [12]. Nevertheless, in the mapping results of myosin, there are only 1–12 AHTP hits. In summary, the bioactivity of fish collagens is highly interesting, especially for their wide applications in the cosmetic industry [50]. 

Scale and skin of fish contain more abundant collagens than muscle, thus the choice of raw materials for collagen preparation is also a challenge [51]. In addition, it is noticeable that the evolutionary rate of platyfish is much higher than any other examined fish species in our phylogenetic analysis (Figure 5 and Figure 6). In our attempt to explore the relationships between water ecology and potential AHTP hit number or mapping ratio of collagens, we could not reach a consistent conclusion. The species with similar habitat niches did not exhibit similar mapping pattern in col4a5 and col8a1, although the closely-related fish species did. Obviously, genetic basis, functional analysis and molecular mechanisms of collagens are worth deep investigation.

### 3.3. Prospective Development of Fish-Derived Antihypertensive Agents

Fishes have always been regarded as omnipotent food resources [15], but it is hard to evaluate the overall nutritional value discrepancy among various fish species. Previous studies reported that marine fish supply proteins equivalent to freshwater fish but with lower fat [52]. In this study, salmon proteins were revealed to have the most abundant AHTPs. From this view, salmons may be a good supplement for treatment of patients with cardiovascular diseases. 

There are many reports with identification of bioactive peptides from salmon proteins, especially from those trimmings [24,28]. Tuna dark muscle have also been suggested as a potential functional food or pharmaceutical for AHTPs with antihypertensive effects [26]. Nevertheless, big-data-based analysis of the whole proteomes from various fish species are rare. This present study supports that fish proteins are good raw materials for further development of antihypertensive agents. Meanwhile, processing methods of fish protein hydrolysates are also ongoing engineering problems for commercial applications. The bioactivity of fish proteins after treatment and manufacture should be considered seriously [25,53].

## 4. Materials and Methods

### 4.1. Data Collection

AHTPs were originally downloaded from the public AHTPDB, which contained 6979 entries (on 28 June 2018). We corrected some confusing records based on published references and deleted those items that had not been well defined. Most IC_50_ values of the AHTPs were confirmed further manually, with retaining of those in the unit of µM. Those duplicated peptides were deleted and the entry for each AHTP was retained with the lowest IC_50_ value. Another 42 sequences were obtained from public literatures of reviews and articles. 

Finally, we compiled 963 peptides in our local database (Table S1). To identify potential AHTPs in fish protein datasets more explicitly and efficiently, we selected the Top 50 AHTPs with the highest inhibitory activity (marked in Table S1), without dipeptides as their high likelihood of random matches, to search against protein datasets. High-quality whole proteome sequences of 17 fish species were downloaded from National Center for Biotechnology Information (NCBI) and Ensemble database, while the grass carp protein dataset was acquired from an exclusively database for this fish in National Center for Gene Research under Chinese Academy of Sciences (Table S2).

### 4.2. Screening for AHTPs

To obtain the target proteins and extract their corresponding matched AHTPs with localization information, we developed a local custom Perl script pipeline to analyze the data. Considering the length discrepancy among sequences, a scoring rule represented by an absolute value was applied to sum the proportion of various AHTPs mapped to a protein. Statistics of total hit number in each mapped protein of the 18 examined fish species and hit number of matched AHTPs in each species were implemented. According to the protein coding gene symbol of each protein sequence, categories of the Top 50 proteins with the most hit number (Hit Top 50) and with the largest scoring value (Score Top 50) were summarized and classified. Collagen subunit proteins were characterized by index terms. Gene symbols of collagens include collagen, persistent plexus, si:dkey-21p1.3, si:dkey-61l1.4, zmp:0000000760, si:dkeyp-44a8.4, si:ch211-196i2.1, and si:dkey-225n22.4.

### 4.3. Construction of Phylogenetic Trees

The collagen subunit protein sequences were collected from homology searches against collected protein datasets and hits of NCBI Blast results. All sequences were further checked in the NCBI and Ensemble database. Accession numbers were provided in Table S7. Those proteins with low-quality were discarded. Multiple sequences of col4a5 and col8a1 were aligned by MAFFT (version 7.037) [54]. Conserved domains were extracted by Gblocks (version 0.91b) [55]. Likelihood-based methods were applied to establish the phylogenetic trees by PhyML (version 3.1) [56] and MrBayes (version 3.2.6) [57]. Models of amino acid replacement were determined by Protest (version 3.4.2) [58]. PhyML analyses were executed under the MtREV+I+G+F (col4a5) and JTT+I+G+F (col8a1) model with bootstrap value of 1000. Markov chains were run for 1,000,000 generations in Bayesian Inference, sampling every 100 generations. The first 2500 convergence runs were discarded as burn-in. Chain stationary and run parameter convergence were further checked using Tracer (version 1.7.1) [59]. Phylogenetic trees were visualized by FigTree (version 1.4.3) [60]. Local visualization of peptides to protein sequences was realized by Perl package “SVG” [61].

## 5. Conclusions

This is the first attempt to develop ATHPs from fish proteins in a high-throughput way. In this study, identification of AHTPs with the highest inhibitory activity from whole proteome datasets of 18 fish species was realized. In addition, the distribution of various AHTPs in protein sequences and protein categories with the most hit number were analyzed. Salmons seem to be a good food resource for people to treat hypertension or improve health, and collagen subunit proteins col4a5 and col8a1 are potent antihypertensive agent substitutes for existence of numerous ATHPs in their sequences. In summary, this is a comprehensive work for discovery of potent AHTPs and their fish proteins/resources at a whole proteome level.

## Figures and Tables

**Table 1:** Categories of proteins with the first top hit and score in 18 examined fish species.

Species	Common Name	The Top Hit	Hit Number	The Top Score	Hit Number
*Latimeria chalumnae*	Coelacanth	Col4a5	89	Col8a1	39
*Lepisosteus oculatus*	Spotted gar	Col4a5	102	Col8a1b	57
*Danio rerio*	Zebrafish	Elastin b	85	Birc2	1
*Ctenopharyngodon idella*	Grass carp	Titin	107	Col8a1	52
*Sinocyclocheilus anshuiensis*	Sa	Titin	95	Unknown ^1^	9
*Sinocyclocheilus rhinocerous*	Sr	Titin	98	Col8a1	61
*Sinocyclocheilus grahami*	Sg	Col4a6	68	Col8a1	59
*Astyanax mexicanus*	Mexican tetra	Col4a5	79	Col8a2	46
*Salmo salar*	Atlantic salmon	Xylanase	122	Xylanase	122
*Gadus morhua*	Atlantic cod	Titin	84	Col8a1	29
*Oryzias latipes*	Medaka	Col4a6	68	Col25a1	24
*Xiphophorus maculatus*	Platyfish	Titin	75	Col8a1a	53
*Gasterosteus aculeatus*	Stickleback	Titin	80	Col8a1	47
*Takifugu rubripes*	Japanese puffer	Col4a5	86	Col8a1a	42
*Tetraodon nigroviridis*	Green spotted puffer	Col4a5	82	Col14a1	5
*Oreochromis niloticus*	Nile tilapia	Col4a6	68	Col25a1	33
*Boleophthalmus pectinirostris*	Bp	Titin	108	Epsin	89
*Periophthalmus magnuspinnatus*	Pm	Col4a5	53	Unknown ^2^	5

^1^ Protein with 74 aa. ^2^ Protein with 71 aa.

## Supplementary Materials

The following are available online at http://www.mdpi.com/1660-3397/16/10/365/s1. Table S1: Established local AHTP database. Table S2: Websites for downloading the protein datasets of 18 fish species. Table S3: Specific alignment results of all mapped proteins and their corresponding matched AHTPs in the 18 examined fish species. Table S4: Statistics of AHTP hit number in each species and comprehensive alignment results in the 18 examined fish species. Table S5: Statistics of hit numbers in each mapped protein from the 18 examined fish species. Table S6: Mapped proteins with the Top 50 scores in the 18 examined fish species. Table S7: Accession numbers of collagen subunit protein sequences and water ecotypes of 33 fish species. Table S8: In situ mapping result of col4a5 from 24 fish species with matched AHTPs. Table S9: In situ mapping result of col8a1 from 28 fish species with matched AHTPs. Table S10: In silico analysis of salmon col8a1.
