# Supplementary material for: High Throughput Identification of Antihypertensive Peptides from Fish Proteome Datasets
Source: Mar Drugs. 2018 Oct 2;16(10):365. doi: 10.3390/md16100365 (PMC6212880; doi:10.3390/md16100365)

**BIOPEP: Profiles of Salmon col8a1 potential biological activity**

| **Protein sequence:** | MVVPPFSAPLPLLVVLLQLAVLPLVHAGAYYRHKQHPQQHQPTPHLSHMGIGGKEQHPQQ HWPGKEMPHMQYPQYRKEIPQMSMHMGKANPHKGGVVNGGQDKGQTIPSGAVGGFPGGLP GEQGPAGPPGPEGPSGPPGPAGEGQPGREGKPGHAGPPGFPGVGKPGLPGIPGKPGSMGE PGVPGELGHSGREGPMGQQGPQGSPGPPGLPGIGKLGAGGLPGQPGPRGDPGHKGLPGLP GLPGPKGDKGIGQPGQPGHKGLAGPPGPPGQGGMPGVGKPGMNGMSGPPGGPGKPGLPGE QGLAGPAGEGGEPGPPGLPGQGKPGQNGLPGQPGMPGGKGHPGPPGFPGKPGLPGFGKPG FPGPKGDKGMGGMPGGPGPKGDKGHGGLPGMLGQPGSIGPAGPLGPMGPPGGLGQPGPKG EAGEGGHKGLPGGQGEPGPIGLTGQNGFPGEGGEPGPRGPTGPVGPQGEGGHKGLPGAPG IPGLPGPKGEGGLPGEKGPQGPKGIPGLGGAGGPIGPPGAPGTKGDSGPPGLPGVDGKGN PGVPGPLGPTGKEGPGGPPGNPGQPGPPGPPGPPGPTDMGAVLPEMGFPPGLDGVKTAGY GKKGKYGGNGGEVMGPNGLEMPAFTALVTTPFPPVGTAVVFDKILYNGRQNYNPQTGVFT CDMPGIYYFAYHISCKGANVWVALMRNDEPVMYTYDEYKKGFLDQASGSAVLPLQPGDTV YLQLPSDQAAGLYAGQYVHSSFSGYLLYPM |
| --- | --- |

| **ID** | **Name of peptide** | **Activity** | **Number** | **Sequence** | **Location** |
| --- | --- | --- | --- | --- | --- |
| 3458 | Prolyl endopeptidase inhibitor | antiamnestic | 1 | GPGG | [572-575] |
| 3459 | Prolyl endopeptidase (PEP) inhibitor | antiamnestic | 19 | PGP | [133-135],[142-144],[211-213],[231-233],[251-253],[274-276],[323-325],[352-354],[374-376],[389-391],[428-430],[451-453],[469-471],[501-503],[562-564],[583-585],[586-588],[589-591],[592-594] |
| 3460 | Prolyl endopeptidase inhibitor | antiamnestic | 78 | PG | [65-66],[118-119],[133-134],[142-143],[150-151],[156-157],[162-163],[165-166],[170-171],[173-174],[176-177],[179-180],[187-188],[190-191],[211-212],[214-215],[217-218],[228-229],[231-232],[237-238],[243-244],[251-252],[262-263],[265-266],[274-275],[277-278],[283-284],[288-289],[297-298],[300-301],[303-304],[306-307],[323-324],[326-327],[329-330],[334-335],[340-341],[343-344],[346-347],[352-353],[355-356],[358-359],[361-362],[364-365],[369-370],[374-375],[386-387],[389-390],[401-402],[407-408],[422-423],[428-429],[445-446],[451-452],[463-464],[469-470],[490-491],[493-494],[498-499],[501-502],[510-511],[522-523],[534-535],[537-538],[546-547],[549-550],[559-560],[562-563],[573-574],[577-578],[580-581],[583-584],[586-587],[589-590],[592-593],[608-609],[686-687],[738-739] |
| 3461 | Prolyl endopeptidase inhibitor | antiamnestic | 47 | GP | [128-129],[131-132],[134-135],[137-138],[140-141],[143-144],[160-161],[200-201],[206-207],[212-213],[232-233],[252-253],[272-273],[275-276],[295-296],[299-300],[315-316],[324-325],[353-354],[375-376],[388-389],[390-391],[411-412],[414-415],[417-418],[420-421],[429-430],[452-453],[470-471],[473-474],[476-477],[479-480],[502-503],[514-515],[517-518],[529-530],[532-533],[544-545],[563-564],[566-567],[572-573],[575-576],[584-585],[587-588],[590-591],[593-594],[635-636] |
| 2653 | ACE inhibitor | ACE inhibitor | 3 | VLP | [21-23],[600-602],[733-735] |
| 2664 | ACE inhibitor | ACE inhibitor | 1 | PLP | [9-11] |
| 3342 | ACE inhibitor | ACE inhibitor | 4 | GPA | [128-130],[143-145],[315-317],[411-413] |
| 3377 | ACE inhibitor (from bovine as1-CN) | ACE inhibitor | 1 | FGK | [366-368] |
| 3381 | ACE inhibitor | ACE inhibitor | 3 | LY | [665-666],[756-757],[771-772] |
| 3383 | ACE inhibitor | ACE inhibitor | 1 | IY | [688-689] |
| 3384 | ACE inhibitor | ACE inhibitor | 2 | VF | [660-661],[678-679] |
| 3388 | ACE inhibitor | ACE inhibitor | 1 | MY | [714-715] |
| 3486 | ACE inhibitor from sake lees | ACE inhibitor | 1 | VW | [702-703] |
| 3502 | ACE inhibitor (BSA fr. 221-222) | ACE inhibitor | 7 | FP | [117-118],[164-165],[357-358],[373-374],[462-463],[606-607],[652-653] |
| 3506 | ACE inhibitor | ACE inhibitor | 8 | GKP | [154-156],[168-170],[177-179],[286-288],[301-303],[332-334],[359-361],[367-369] |
| 3524 | ACE inhibitor (from bovine beta-CN) | ACE inhibitor | 1 | VPP | [3-5] |
| 3532 | ACE inhibitor | ACE inhibitor | 2 | GY | [617-618],[768-769] |
| 3537 | ACE inhibitor | ACE inhibitor | 2 | PR | [233-234],[471-472] |
| 3542 | ACE inhibitor | ACE inhibitor | 1 | LQP | [736-738] |
| 3550 | ACE inhibitor (from bovine beta-Lg) | ACE inhibitor | 2 | YL | [745-746],[769-770] |
| 3553 | ACE inhibitor | ACE inhibitor | 1 | YG | [626-627] |
| 3563 | ACE inhibitor | ACE inhibitor | 2 | AY | [29-30],[692-693] |
| 3666 | ACE inhibitor | ACE inhibitor | 2 | YP | [74-75],[772-773] |
| 7506 | ACE inhibitor from Alaskan pollack skin | ACE inhibitor | 2 | GPL | [414-416],[563-565] |
| 7507 | ACE inhibitor from Alaskan pollack skin | ACE inhibitor | 10 | PGL | [170-172],[214-216],[243-245],[303-305],[326-328],[361-363],[498-500],[522-524],[546-548],[608-610] |
| 7508 | ACE inhibitor from Alaskan pollack skin | ACE inhibitor | 2 | LGP | [416-418],[565-567] |
| 7509 | ACE inhibitor from Alaskan pollack skin | ACE inhibitor | 17 | GLP | [120-122],[171-173],[215-217],[226-228],[241-243],[244-246],[249-251],[304-306],[327-329],[338-340],[362-364],[399-401],[443-445],[488-490],[499-501],[508-510],[547-549] |
| 7510 | ACE inhibitor from Alaskan pollack skin | ACE inhibitor | 2 | PLG | [415-417],[564-566] |
| 7511 | ACE inhibitor from Alaskan pollack skin | ACE inhibitor | 15 | LPG | [172-174],[216-218],[227-229],[242-244],[250-252],[305-307],[328-330],[339-341],[363-365],[400-402],[444-446],[489-491],[500-502],[509-511],[548-550] |
| 7512 | ACE inhibitor from Alaskan pollack skin | ACE inhibitor | 47 | GP | [128-129],[131-132],[134-135],[137-138],[140-141],[143-144],[160-161],[200-201],[206-207],[212-213],[232-233],[252-253],[272-273],[275-276],[295-296],[299-300],[315-316],[324-325],[353-354],[375-376],[388-389],[390-391],[411-412],[414-415],[417-418],[420-421],[429-430],[452-453],[470-471],[473-474],[476-477],[479-480],[502-503],[514-515],[517-518],[529-530],[532-533],[544-545],[563-564],[566-567],[572-573],[575-576],[584-585],[587-588],[590-591],[593-594],[635-636] |
| 7513 | ACE inhibitor from Alaskan pollack skin | ACE inhibitor | 6 | PL | [9-10],[11-12],[23-24],[415-416],[564-565],[735-736] |
| 7545 | ACE inhibitor | ACE inhibitor | 1 | GPV | [476-478] |
| 7550 | ACE inhibitor | ACE inhibitor | 1 | LYP | [771-773] |
| 7554 | ACE inhibitor from Tricholoma giganteum | ACE inhibitor | 3 | GEP | [321-323],[449-451],[467-469] |
| 7558 | ACE inhibitor from buckwheat | ACE inhibitor | 1 | VK | [613-614] |
| 7581 | ACE inhibitor | ACE inhibitor | 5 | IP | [81-82],[109-110],[175-176],[497-498],[521-522] |
| 7583 | ACE inhibitor | ACE inhibitor | 1 | AF | [643-644] |
| 7584 | ACE inhibitor | ACE inhibitor | 3 | AP | [8-9],[492-493],[536-537] |
| 7585 | ACE inhibitor | ACE inhibitor | 3 | LA | [19-20],[270-271],[313-314] |
| 7587 | ACE inhibitor | ACE inhibitor | 3 | VP | [3-4],[189-190],[561-562] |
| 7589 | ACE inhibitor | ACE inhibitor | 1 | YA | [757-758] |
| 7590 | ACE inhibitor | ACE inhibitor | 1 | AA | [753-754] |
| 7591 | ACE inhibitor | ACE inhibitor | 7 | GF | [116-117],[163-164],[356-357],[365-366],[461-462],[605-606],[723-724] |
| 7594 | ACE inhibitor | ACE inhibitor | 5 | VG | [114-115],[167-168],[285-286],[478-479],[655-656] |
| 7595 | ACE inhibitor | ACE inhibitor | 6 | IG | [51-52],[219-220],[259-260],[410-411],[454-455],[531-532] |
| 7596 | ACE inhibitor | ACE inhibitor | 6 | GI | [50-51],[174-175],[218-219],[258-259],[520-521],[687-688] |
| 7597 | ACE inhibitor | ACE inhibitor | 7 | GM | [281-282],[289-290],[292-293],[344-345],[381-382],[384-385],[402-403] |
| 7598 | ACE inhibitor | ACE inhibitor | 8 | GA | [28-29],[112-113],[223-224],[491-492],[526-527],[535-536],[598-599],[699-700] |
| 7599 | ACE inhibitor | ACE inhibitor | 25 | GL | [120-121],[171-172],[215-216],[226-227],[241-242],[244-245],[249-250],[269-270],[304-305],[312-313],[327-328],[338-339],[362-363],[399-400],[424-425],[443-444],[455-456],[488-489],[499-500],[508-509],[523-524],[547-548],[609-610],[638-639],[755-756] |
| 7600 | ACE inhibitor | ACE inhibitor | 14 | AG | [27-28],[130-131],[145-146],[159-160],[224-225],[271-272],[314-315],[317-318],[413-414],[436-437],[527-528],[616-617],[754-755],[758-759] |
| 7601 | ACE inhibitor | ACE inhibitor | 8 | GH | [157-158],[194-195],[238-239],[266-267],[350-351],[396-397],[440-441],[485-486] |
| 7602 | ACE inhibitor | ACE inhibitor | 1 | HL | [45-46] |
| 7603 | ACE inhibitor | ACE inhibitor | 3 | GR | [151-152],[197-198],[668-669] |
| 7604 | ACE inhibitor | ACE inhibitor | 22 | KG | [95-96],[105-106],[240-241],[254-255],[257-258],[268-269],[349-350],[377-378],[380-381],[392-393],[395-396],[431-432],[442-443],[487-488],[504-505],[513-514],[519-520],[540-541],[554-555],[623-624],[698-699],[722-723] |
| 7605 | ACE inhibitor | ACE inhibitor | 1 | FG | [366-367] |
| 7607 | ACE inhibitor | ACE inhibitor | 4 | GS | [180-181],[209-210],[408-409],[730-731] |
| 7608 | ACE inhibitor | ACE inhibitor | 8 | GV | [97-98],[166-167],[188-189],[284-285],[550-551],[560-561],[612-613],[677-678] |
| 7609 | ACE inhibitor | ACE inhibitor | 9 | MG | [49-50],[88-89],[182-183],[202-203],[382-383],[419-420],[597-598],[604-605],[634-635] |
| 7610 | ACE inhibitor | ACE inhibitor | 17 | GQ | [102-103],[106-107],[148-149],[203-204],[229-230],[260-261],[263-264],[278-279],[330-331],[335-336],[341-342],[405-406],[426-427],[447-448],[458-459],[581-582],[759-760] |
| 7611 | ACE inhibitor | ACE inhibitor | 17 | GK | [53-54],[66-67],[89-90],[154-155],[168-169],[177-178],[220-221],[286-287],[301-302],[332-333],[348-349],[359-360],[367-368],[553-554],[569-570],[621-622],[624-625] |
| 7612 | ACE inhibitor | ACE inhibitor | 2 | GT | [538-539],[656-657] |
| 7614 | ACE inhibitor | ACE inhibitor | 1 | HG | [397-398] |
| 7615 | ACE inhibitor | ACE inhibitor | 15 | GE | [125-126],[146-147],[183-184],[191-192],[307-308],[318-319],[321-322],[437-438],[449-450],[464-465],[467-468],[482-483],[505-506],[511-512],[631-632] |
| 7616 | ACE inhibitor | ACE inhibitor | 24 | GG | [52-53],[96-97],[101-102],[115-116],[119-120],[225-226],[280-281],[298-299],[320-321],[347-348],[383-384],[387-388],[398-399],[423-424],[439-440],[446-447],[466-467],[484-485],[507-508],[525-526],[528-529],[574-575],[627-628],[630-631] |
| 7617 | ACE inhibitor | ACE inhibitor | 9 | QG | [127-128],[205-206],[208-209],[279-280],[311-312],[331-332],[448-449],[481-482],[516-517] |
| 7618 | ACE inhibitor | ACE inhibitor | 7 | SG | [111-112],[139-140],[196-197],[294-295],[543-544],[729-730],[767-768] |
| 7619 | ACE inhibitor | ACE inhibitor | 7 | LG | [193-194],[222-223],[404-405],[416-417],[425-426],[524-525],[565-566] |
| 7620 | ACE inhibitor | ACE inhibitor | 6 | GD | [235-236],[255-256],[378-379],[393-394],[541-542],[739-740] |
| 7621 | ACE inhibitor | ACE inhibitor | 4 | TG | [457-458],[475-476],[568-569],[676-677] |
| 7622 | ACE inhibitor | ACE inhibitor | 10 | EG | [136-137],[147-148],[153-154],[199-200],[319-320],[438-439],[465-466],[483-484],[506-507],[571-572] |
| 7623 | ACE inhibitor | ACE inhibitor | 1 | EA | [435-436] |
| 7624 | ACE inhibitor | ACE inhibitor | 7 | NG | [100-101],[291-292],[337-338],[460-461],[629-630],[637-638],[667-668] |
| 7625 | ACE inhibitor | ACE inhibitor | 78 | PG | [65-66],[118-119],[133-134],[142-143],[150-151],[156-157],[162-163],[165-166],[170-171],[173-174],[176-177],[179-180],[187-188],[190-191],[211-212],[214-215],[217-218],[228-229],[231-232],[237-238],[243-244],[251-252],[262-263],[265-266],[274-275],[277-278],[283-284],[288-289],[297-298],[300-301],[303-304],[306-307],[323-324],[326-327],[329-330],[334-335],[340-341],[343-344],[346-347],[352-353],[355-356],[358-359],[361-362],[364-365],[369-370],[374-375],[386-387],[389-390],[401-402],[407-408],[422-423],[428-429],[445-446],[451-452],[463-464],[469-470],[490-491],[493-494],[498-499],[501-502],[510-511],[522-523],[534-535],[537-538],[546-547],[549-550],[559-560],[562-563],[573-574],[577-578],[580-581],[583-584],[586-587],[589-590],[592-593],[608-609],[686-687],[738-739] |
| 7647 | ACE inhibitor | ACE inhibitor | 1 | YGG | [626-628] |
| 7681 | ACE inhibitor from soy | ACE inhibitor | 2 | DG | [552-553],[611-612] |
| 7682 | ACE inhibitor from garlic | ACE inhibitor | 1 | NY | [671-672] |
| 7685 | ACE inhibitor from garlic | ACE inhibitor | 1 | SF | [765-766] |
| 7691 | ACE inhibitor from wakame | ACE inhibitor | 1 | KY | [625-626] |
| 7693 | ACE inhibitor from wakame | ACE inhibitor | 1 | KL | [221-222] |
| 7697 | ACE inhibitor from wakame | ACE inhibitor | 1 | YK | [720-721] |
| 7743 | ACE inhibitor | ACE inhibitor | 1 | KA | [90-91] |
| 7752 | ACE inhibitor from shark meat hydrolysate | ACE inhibitor | 1 | EY | [719-720] |
| 7810 | ACE inhibitor from anchovy and bonito | ACE inhibitor | 8 | KP | [155-156],[169-170],[178-179],[287-288],[302-303],[333-334],[360-361],[368-369] |
| 7820 | ACE inhibitor from wheat gliadin | ACE inhibitor | 16 | GPP | [131-133],[140-142],[160-162],[212-214],[272-274],[275-277],[295-297],[324-326],[353-355],[420-422],[532-534],[544-546],[575-577],[584-586],[587-589],[590-592] |
| 7826 | ACE inhibitor | ACE inhibitor | 1 | EI | [80-81] |
| 7828 | ACE inhibitor | ACE inhibitor | 1 | EV | [632-633] |
| 7831 | ACE inhibitor | ACE inhibitor | 3 | LQ | [17-18],[736-737],[746-747] |
| 7833 | ACE inhibitor | ACE inhibitor | 4 | PT | [42-43],[474-475],[567-568],[594-595] |
| 7836 | ACE inhibitor | ACE inhibitor | 19 | PP | [4-5],[132-133],[141-142],[161-162],[213-214],[273-274],[276-277],[296-297],[325-326],[354-355],[421-422],[533-534],[545-546],[576-577],[585-586],[588-589],[591-592],[607-608],[653-654] |
| 7837 | ACE inhibitor | ACE inhibitor | 8 | PQ | [37-38],[58-59],[75-76],[82-83],[207-208],[480-481],[515-516],[674-675] |
| 7840 | ACE inhibitor | ACE inhibitor | 1 | EK | [512-513] |
| 7841 | ACE inhibitor | ACE inhibitor | 4 | KE | [54-55],[67-68],[79-80],[570-571] |
| 7842 | ACE inhibitor | ACE inhibitor | 3 | HP | [36-37],[57-58],[351-352] |
| 7843 | ACE inhibitor | ACE inhibitor | 3 | PH | [44-45],[70-71],[93-94] |
| 7844 | ACE inhibitor | ACE inhibitor | 6 | HK | [33-34],[94-95],[239-240],[267-268],[441-442],[486-487] |
| 8308 | ACE inhibitor | ACE inhibitor | 1 | VVPP | [2-5] |
| 8951 | ACE inhibitor | ACE inhibitor | 5 | AV | [20-21],[113-114],[599-600],[658-659],[732-733] |
| 9033 | ACE inhibitor | ACE inhibitor | 1 | GLY | [755-757] |
| 9034 | ACE inhibitor | ACE inhibitor | 8 | GQP | [148-150],[229-231],[260-262],[263-265],[341-343],[405-407],[426-428],[581-583] |
| 9041 | ACE inhibitor | ACE inhibitor | 5 | AGP | [130-132],[159-161],[271-273],[314-316],[413-415] |
| 9044 | ACE inhibitor | ACE inhibitor | 1 | VVF | [659-661] |
| 9049 | ACE inhibitor | ACE inhibitor | 1 | FDK | [661-663] |
| 9052 | ACE inhibitor | ACE inhibitor | 2 | GPM | [200-202],[417-419] |
| 9060 | ACE inhibitor | ACE inhibitor | 3 | AVL | [20-22],[599-601],[732-734] |
| 9069 | ACE inhibitor | ACE inhibitor | 1 | VGP | [478-480] |
| 9073 | ACE inhibitor | ACE inhibitor | 2 | TP | [43-44],[650-651] |
| 9075 | ACE inhibitor | ACE inhibitor | 2 | DM | [596-597],[684-685] |
| 9077 | ACE inhibitor | ACE inhibitor | 1 | YV | [761-762] |
| 9079 | ACE inhibitor | ACE inhibitor | 1 | IL | [664-665] |
| 9087 | ACE inhibitor | ACE inhibitor | 1 | YH | [693-694] |
| 9142 | ACE inhibitor | ACE inhibitor | 2 | MGP | [419-421],[634-636] |
| 9145 | ACE inhibitor | ACE inhibitor | 3 | SGP | [139-141],[294-296],[543-545] |
| 9146 | ACE inhibitor | ACE inhibitor | 3 | QGP | [127-129],[205-207],[516-518] |
| 9148 | ACE inhibitor | ACE inhibitor | 1 | KGP | [513-515] |
| 9151 | ACE inhibitor | ACE inhibitor | 1 | TGP | [475-477] |
| 9155 | ACE inhibitor | ACE inhibitor | 1 | EQGP | [126-129] |
| 9157 | ACE inhibitor | ACE inhibitor | 1 | EKGP | [512-515] |
| 9173 | ACE inhibitor | ACE inhibitor | 2 | RG | [234-235],[472-473] |
| 9185 | ACE inhibitor | ACE inhibitor | 2 | YN | [666-667],[672-673] |
| 9191 | ACE inhibitor | ACE inhibitor | 1 | GHG | [396-398] |
| 9196 | ACE inhibitor | ACE inhibitor | 1 | AVV | [658-660] |
| 9242 | ACE inhibitor | ACE inhibitor | 1 | PLPLL | [9-13] |
| 3040 | Inhibitor of fibrinogen and thrombin coagulation | antithrombotic | 2 | GPRG | [232-235],[470-473] |
| 3041 | inhibitor of fibrinogen and thrombin coagulation | antithrombotic | 1 | GPRGP | [470-474] |
| 3047 | inhibitor of fibrinogen and thrombin coagulation | antithrombotic | 2 | GPR | [232-234],[470-472] |
| 3283 | Antithrombotic peptide | antithrombotic | 47 | GP | [128-129],[131-132],[134-135],[137-138],[140-141],[143-144],[160-161],[200-201],[206-207],[212-213],[232-233],[252-253],[272-273],[275-276],[295-296],[299-300],[315-316],[324-325],[353-354],[375-376],[388-389],[390-391],[411-412],[414-415],[417-418],[420-421],[429-430],[452-453],[470-471],[473-474],[476-477],[479-480],[502-503],[514-515],[517-518],[529-530],[532-533],[544-545],[563-564],[566-567],[572-573],[575-576],[584-585],[587-588],[590-591],[593-594],[635-636] |
| 3284 | antithrombotic peptide | antithrombotic | 19 | PGP | [133-135],[142-144],[211-213],[231-233],[251-253],[274-276],[323-325],[352-354],[374-376],[389-391],[428-430],[451-453],[469-471],[501-503],[562-564],[583-585],[586-588],[589-591],[592-594] |
| 3285 | Antithrombotic peptide | antithrombotic | 78 | PG | [65-66],[118-119],[133-134],[142-143],[150-151],[156-157],[162-163],[165-166],[170-171],[173-174],[176-177],[179-180],[187-188],[190-191],[211-212],[214-215],[217-218],[228-229],[231-232],[237-238],[243-244],[251-252],[262-263],[265-266],[274-275],[277-278],[283-284],[288-289],[297-298],[300-301],[303-304],[306-307],[323-324],[326-327],[329-330],[334-335],[340-341],[343-344],[346-347],[352-353],[355-356],[358-359],[361-362],[364-365],[369-370],[374-375],[386-387],[389-390],[401-402],[407-408],[422-423],[428-429],[445-446],[451-452],[463-464],[469-470],[490-491],[493-494],[498-499],[501-502],[510-511],[522-523],[534-535],[537-538],[546-547],[549-550],[559-560],[562-563],[573-574],[577-578],[580-581],[583-584],[586-587],[589-590],[592-593],[608-609],[686-687],[738-739] |
| 3462 |  | antithrombotic | 1 | GPGG | [572-575] |
| 2882 | Immunostimulating peptide | immunomodulating | 1 | YG | [626-627] |
| 3741 | Immunomodulating peptide | immunomodulating | 1 | YGG | [626-628] |
| 3094 | gliadin 1 exorphin | opioid | 2 | PLG | [415-417],[564-566] |
| 8320 | Glucose uptake stimulating peptide | stimulating | 4 | VL | [15-16],[21-22],[600-601],[733-734] |
| 8321 | Glucose uptake stimulating peptide | stimulating | 3 | LV | [13-14],[24-25],[647-648] |
| 8323 | Glucose uptake stimulating peptide | stimulating | 1 | IL | [664-665] |
| 8326 | Glucose uptake stimulating peptide | stimulating | 3 | LL | [12-13],[16-17],[770-771] |
| 3061 | Immunostimulating peptide | immunostimulating | 1 | GFL | [723-725] |
| 3065 | fragment of bovine beta-casein 191-193 | immunostimulating | 1 | LLY | [770-772] |
| 2890 | neuropeptide | neuropeptide | 17 | GQ | [102-103],[106-107],[148-149],[203-204],[229-230],[260-261],[263-264],[278-279],[330-331],[335-336],[341-342],[405-406],[426-427],[447-448],[458-459],[581-582],[759-760] |
| 8310 | Anxiolytic peptide | neuropeptide | 2 | YL | [745-746],[769-770] |
| 2737 | Peptide regulating phosphoinositol metabolism | regulating | 1 | GFL | [723-725] |
| 2739 | Peptide regulating phosphoinositol metabolism | regulating | 1 | GLY | [755-757] |
| 2753 | peptide regulating the stomach mucosal membrane activity | regulating | 47 | GP | [128-129],[131-132],[134-135],[137-138],[140-141],[143-144],[160-161],[200-201],[206-207],[212-213],[232-233],[252-253],[272-273],[275-276],[295-296],[299-300],[315-316],[324-325],[353-354],[375-376],[388-389],[390-391],[411-412],[414-415],[417-418],[420-421],[429-430],[452-453],[470-471],[473-474],[476-477],[479-480],[502-503],[514-515],[517-518],[529-530],[532-533],[544-545],[563-564],[566-567],[572-573],[575-576],[584-585],[587-588],[590-591],[593-594],[635-636] |
| 2754 | peptide regulating the stomach mucosal membrane activity | regulating | 78 | PG | [65-66],[118-119],[133-134],[142-143],[150-151],[156-157],[162-163],[165-166],[170-171],[173-174],[176-177],[179-180],[187-188],[190-191],[211-212],[214-215],[217-218],[228-229],[231-232],[237-238],[243-244],[251-252],[262-263],[265-266],[274-275],[277-278],[283-284],[288-289],[297-298],[300-301],[303-304],[306-307],[323-324],[326-327],[329-330],[334-335],[340-341],[343-344],[346-347],[352-353],[355-356],[358-359],[361-362],[364-365],[369-370],[374-375],[386-387],[389-390],[401-402],[407-408],[422-423],[428-429],[445-446],[451-452],[463-464],[469-470],[490-491],[493-494],[498-499],[501-502],[510-511],[522-523],[534-535],[537-538],[546-547],[549-550],[559-560],[562-563],[573-574],[577-578],[580-581],[583-584],[586-587],[589-590],[592-593],[608-609],[686-687],[738-739] |
| 2755 | peptide regulating the stomach mucosal membrane activity | regulating | 1 | GPGG | [572-575] |
| 2756 | peptide regulating the stomach mucosal membrane activity | regulating | 19 | PGP | [133-135],[142-144],[211-213],[231-233],[251-253],[274-276],[323-325],[352-354],[374-376],[389-391],[428-430],[451-453],[469-471],[501-503],[562-564],[583-585],[586-588],[589-591],[592-594] |
| 8319 | Dvl protein binding | anticancer | 1 | VWV | [702-704] |
| 3317 |  | antioxidative | 1 | HL | [45-46] |
| 7866 | peptide from Okara protein | antioxidative | 2 | AY | [29-30],[692-693] |
| 7872 | peptide from soybean protein isolates: beta-conglycinin and glycinin | antioxidative | 3 | LY | [665-666],[756-757],[771-772] |
| 7873 | peptide from soybean protein isolates: beta-conglycinin and glycinin | antioxidative | 1 | IY | [688-689] |
| 7888 | antioxidative peptide | antioxidative | 1 | EL | [192-193] |
| 7938 | synthetic peptide | antioxidative | 1 | YYR | [30-32] |
| 7943 | synthetic peptide | antioxidative | 1 | YYF | [689-691] |
| 7953 | synthetic peptide | antioxidative | 1 | AYY | [29-31] |
| 7954 | synthetic peptide | antioxidative | 1 | IYY | [688-690] |
| 7973 | synthetic peptide | antioxidative | 1 | YTY | [715-717] |
| 8028 | synthetic peptide | antioxidative | 1 | PHK | [93-95] |
| 8029 | synthetic peptide | antioxidative | 1 | PHL | [44-46] |
| 8030 | synthetic peptide | antioxidative | 1 | PHM | [70-72] |
| 8060 | synthetic peptide | antioxidative | 1 | RHK | [32-34] |
| 8090 | peptide derived from sardine muscle | antioxidative | 1 | MY | [714-715] |
| 8114 | peptide derived from sardinelle by-products proteins (Sardinella aurita) | antioxidative | 3 | GGE | [320-322],[466-468],[630-632] |
| 8218 | Antioxidative peptide | antioxidative | 8 | KP | [155-156],[169-170],[178-179],[287-288],[302-303],[333-334],[360-361],[368-369] |
| 8219 | antioxidative peptide | antioxidative | 1 | TY | [716-717] |
| 8461 | Antioxidant peptide from marine bivalve (Mactra veneriformis) | antioxidative | 1 | VW | [702-703] |
| 8987 | Antioxidative peptide | antioxidative | 16 | GPP | [131-133],[140-142],[160-162],[212-214],[272-274],[275-277],[295-297],[324-326],[353-355],[420-422],[532-534],[544-546],[575-577],[584-586],[587-589],[590-592] |
| 9179 | Antioxidative peptide | antioxidative | 1 | QYP | [73-75] |
| 9349 | Antioxidative peptide | antioxidative | 1 | YLL | [769-771] |
| 3751 |  | bacterial permease ligand | 2 | KK | [622-623],[721-722] |
| 3782 | dipeptidyl carboxypeptidase inhibitor | inhibitor | 6 | PPGP | [132-135],[141-144],[273-276],[585-588],[588-591],[591-594] |
| 3464 | Chemotactic peptide | chemotactic | 19 | PGP | [133-135],[142-144],[211-213],[231-233],[251-253],[274-276],[323-325],[352-354],[374-376],[389-391],[428-430],[451-453],[469-471],[501-503],[562-564],[583-585],[586-588],[589-591],[592-594] |
| 3046 | inhibitor of insulin secretion | anorectic | 19 | PGP | [133-135],[142-144],[211-213],[231-233],[251-253],[274-276],[323-325],[352-354],[374-376],[389-391],[428-430],[451-453],[469-471],[501-503],[562-564],[583-585],[586-588],[589-591],[592-594] |
| 3164 | laminin-like peptide | embryotoxic | 1 | RGD | [234-236] |
| 4006 | Ubiqitin-mediated proteolysis activating peptide | activating ubiquitin-mediated proteolysis | 3 | LA | [19-20],[270-271],[313-314] |
| 9387 | Alpha-glucosidase inhibitor | alpha-glucosidase inhibitor | 1 | VW | [702-703] |
| 9383 | HMG-CoA reductase inhibitor | HMG-CoA reductase inhibitor | 1 | GGV | [96-98] |
| 3169 | dipeptidyl peptidase IV inhibitor (DPP IV inhibitor) | dipeptidyl peptidase IV inhibitor | 47 | GP | [128-129],[131-132],[134-135],[137-138],[140-141],[143-144],[160-161],[200-201],[206-207],[212-213],[232-233],[252-253],[272-273],[275-276],[295-296],[299-300],[315-316],[324-325],[353-354],[375-376],[388-389],[390-391],[411-412],[414-415],[417-418],[420-421],[429-430],[452-453],[470-471],[473-474],[476-477],[479-480],[502-503],[514-515],[517-518],[529-530],[532-533],[544-545],[563-564],[566-567],[572-573],[575-576],[584-585],[587-588],[590-591],[593-594],[635-636] |
| 3170 | dipeptidyl peptidase IV inhibitor (DPP IV inhibitor) | dipeptidyl peptidase IV inhibitor | 19 | PP | [4-5],[132-133],[141-142],[161-162],[213-214],[273-274],[276-277],[296-297],[325-326],[354-355],[421-422],[533-534],[545-546],[576-577],[585-586],[588-589],[591-592],[607-608],[653-654] |
| 3171 | dipeptidyl peptidase IV inhibitor (DPP IV inhibitor) | dipeptidyl peptidase IV inhibitor | 6 | MP | [69-70],[282-283],[345-346],[385-386],[641-642],[685-686] |
| 3172 | dipeptidyl peptidase IV inhibitor (DPP IV inhibitor) | dipeptidyl peptidase IV inhibitor | 1 | VA | [704-705] |
| 3174 | dipeptidyl peptidase IV inhibitor (DPP IV inhibitor) | dipeptidyl peptidase IV inhibitor | 1 | KA | [90-91] |
| 3175 | dipeptidyl peptidase IV inhibitor (DPP IV inhibitor) | dipeptidyl peptidase IV inhibitor | 3 | LA | [19-20],[270-271],[313-314] |
| 3176 | dipeptidyl peptidase IV inhibitor (DPP IV inhibitor) | dipeptidyl peptidase IV inhibitor | 1 | FA | [691-692] |
| 3177 | dipeptidyl peptidase IV inhibitor (DPP IV inhibitor) | dipeptidyl peptidase IV inhibitor | 3 | AP | [8-9],[492-493],[536-537] |
| 3179 | dipeptidyl peptidase IV inhibitor (DPP IV inhibitor) | dipeptidyl peptidase IV inhibitor | 5 | PA | [129-130],[144-145],[316-317],[412-413],[642-643] |
| 3180 | dipeptidyl peptidase IV inhibitor (DPP IV inhibitor) | dipeptidyl peptidase IV inhibitor | 22 | LP | [10-11],[22-23],[121-122],[172-173],[216-217],[227-228],[242-243],[245-246],[250-251],[305-306],[328-329],[339-340],[363-364],[400-401],[444-445],[489-490],[500-501],[509-510],[548-549],[601-602],[734-735],[748-749] |
| 3181 | dipeptidyl peptidase IV inhibitor (DPP IV inhibitor) | dipeptidyl peptidase IV inhibitor | 3 | VP | [3-4],[189-190],[561-562] |
| 3182 | dipeptidyl peptidase IV inhibitor (DPP IV inhibitor) | dipeptidyl peptidase IV inhibitor | 3 | LL | [12-13],[16-17],[770-771] |
| 3183 | dipeptidyl peptidase IV inhibitor (DPP IV inhibitor) | dipeptidyl peptidase IV inhibitor | 4 | VV | [2-3],[14-15],[98-99],[659-660] |
| 3184 | dipeptidyl peptidase IV inhibitor (DPP IV inhibitor) | dipeptidyl peptidase IV inhibitor | 2 | HA | [26-27],[158-159] |
| 8498 | dipeptidyl peptidase IV inhibitor (DPP IV inhibitor) | dipeptidyl peptidase IV inhibitor | 17 | GQ | [102-103],[106-107],[148-149],[203-204],[229-230],[260-261],[263-264],[278-279],[330-331],[335-336],[341-342],[405-406],[426-427],[447-448],[458-459],[581-582],[759-760] |
| 8500 | dipeptidyl peptidase IV inhibitor (DPP IV inhibitor) | dipeptidyl peptidase IV inhibitor | 2 | APG | [492-494],[536-538] |
| 8501 | Dipeptidyl peptidase IV inhibitor (DPP IV inhibitor) | dipeptidyl peptidase IV inhibitor | 5 | IP | [81-82],[109-110],[175-176],[497-498],[521-522] |
| 8503 | Dipeptidyl peptidase IV inhibitor (DPP IV inhibitor) | dipeptidyl peptidase IV inhibitor | 2 | TP | [43-44],[650-651] |
| 8504 | Dipeptidyl peptidase IV inhibitor (DPP IV inhibitor) | dipeptidyl peptidase IV inhibitor | 1 | WP | [64-65] |
| 8505 | Dipeptidyl peptidase IV inhibitor (DPP IV inhibitor) | dipeptidyl peptidase IV inhibitor | 1 | SP | [210-211] |
| 8506 | dipeptidyl peptidase IV inhibitor (DPP IV inhibitor) | dipeptidyl peptidase IV inhibitor | 7 | FP | [117-118],[164-165],[357-358],[373-374],[462-463],[606-607],[652-653] |
| 8519 | dipeptidyl peptidase IV inhibitor (DPP IV inhibitor) | dipeptidyl peptidase IV inhibitor | 8 | KP | [155-156],[169-170],[178-179],[287-288],[302-303],[333-334],[360-361],[368-369] |
| 8520 | dipeptidyl peptidase IV inhibitor (DPP IV inhibitor) | dipeptidyl peptidase IV inhibitor | 3 | HP | [36-37],[57-58],[351-352] |
| 8521 | dipeptidyl peptidase IV inhibitor (DPP IV inhibitor) | dipeptidyl peptidase IV inhibitor | 2 | YP | [74-75],[772-773] |
| 8522 | dipeptidyl peptidase IV inhibitor (DPP IV inhibitor) | dipeptidyl peptidase IV inhibitor | 4 | GPA | [128-130],[143-145],[315-317],[411-413] |
| 8524 | dipeptidyl peptidase IV inhibitor (DPP IV inhibitor) | dipeptidyl peptidase IV inhibitor | 8 | GA | [28-29],[112-113],[223-224],[491-492],[526-527],[535-536],[598-599],[699-700] |
| 8529 | dipeptidyl peptidase IV inhibitor (DPP IV inhibitor) | dipeptidyl peptidase IV inhibitor | 4 | EP | [322-323],[450-451],[468-469],[711-712] |
| 8530 | dipeptidyl peptidase IV inhibitor (DPP IV inhibitor) | dipeptidyl peptidase IV inhibitor | 3 | NP | [92-93],[579-580],[673-674] |
| 8531 | dipeptidyl peptidase IV inhibitor (DPP IV inhibitor) | dipeptidyl peptidase IV inhibitor | 3 | TA | [615-616],[645-646],[657-658] |
| 8532 | dipeptidyl peptidase IV inhibitor (DPP IV inhibitor) | dipeptidyl peptidase IV inhibitor | 10 | QP | [41-42],[149-150],[230-231],[261-262],[264-265],[342-343],[406-407],[427-428],[582-583],[737-738] |
| 8555 | dipeptidyl peptidase IV inhibitor (DPP IV inhibitor) | dipeptidyl peptidase IV inhibitor | 1 | FL | [724-725] |
| 8556 | dipeptidyl peptidase IV inhibitor (DPP IV inhibitor) | dipeptidyl peptidase IV inhibitor | 1 | WV | [703-704] |
| 8557 | dipeptidyl peptidase IV inhibitor (DPP IV inhibitor) | dipeptidyl peptidase IV inhibitor | 1 | HL | [45-46] |
| 8558 | dipeptidyl peptidase IV inhibitor (DPP IV inhibitor) | dipeptidyl peptidase IV inhibitor | 1 | EK | [512-513] |
| 8559 | dipeptidyl peptidase IV inhibitor (DPP IV inhibitor) | dipeptidyl peptidase IV inhibitor | 2 | AL | [646-647],[705-706] |
| 8561 | dipeptidyl peptidase IV inhibitor (DPP IV inhibitor) | dipeptidyl peptidase IV inhibitor | 25 | GL | [120-121],[171-172],[215-216],[226-227],[241-242],[244-245],[249-250],[269-270],[304-305],[312-313],[327-328],[338-339],[362-363],[399-400],[424-425],[443-444],[455-456],[488-489],[499-500],[508-509],[523-524],[547-548],[609-610],[638-639],[755-756] |
| 8616 | dipeptidyl peptidase IV inhibitor (DPP IV inhibitor) | dipeptidyl peptidase IV inhibitor | 3 | LPL | [10-12],[22-24],[734-736] |
| 8637 | dipeptidyl peptidase IV inhibitor (DPP IV inhibitor) | dipeptidyl peptidase IV inhibitor | 1 | AA | [753-754] |
| 8638 | dipeptidyl peptidase IV inhibitor (DPP IV inhibitor) | dipeptidyl peptidase IV inhibitor | 6 | PL | [9-10],[11-12],[23-24],[415-416],[564-565],[735-736] |
| 8653 | dipeptidyl peptidase IV inhibitor (DPP IV inhibitor) | dipeptidyl peptidase IV inhibitor | 17 | PPG | [132-134],[141-143],[161-163],[213-215],[273-275],[276-278],[296-298],[325-327],[354-356],[421-423],[533-535],[545-547],[576-578],[585-587],[588-590],[591-593],[607-609] |
| 8689 | dipeptidyl peptidase IV inhibitor (DPP IV inhibitor) | dipeptidyl peptidase IV inhibitor | 1 | LQP | [736-738] |
| 8696 | dipeptidyl peptidase IV inhibitor (DPP IV inhibitor) | dipeptidyl peptidase IV inhibitor | 1 | YT | [715-716] |
| 8759 | dipeptidyl peptidase IV inhibitor (DPP IV inhibitor) | dipeptidyl peptidase IV inhibitor | 1 | AF | [643-644] |
| 8760 | dipeptidyl peptidase IV inhibitor (DPP IV inhibitor) | dipeptidyl peptidase IV inhibitor | 14 | AG | [27-28],[130-131],[145-146],[159-160],[224-225],[271-272],[314-315],[317-318],[413-414],[436-437],[527-528],[616-617],[754-755],[758-759] |
| 8762 | dipeptidyl peptidase IV inhibitor (DPP IV inhibitor) | dipeptidyl peptidase IV inhibitor | 1 | AS | [728-729] |
| 8764 | dipeptidyl peptidase IV inhibitor (DPP IV inhibitor) | dipeptidyl peptidase IV inhibitor | 5 | AV | [20-21],[113-114],[599-600],[658-659],[732-733] |
| 8765 | dipeptidyl peptidase IV inhibitor (DPP IV inhibitor) | dipeptidyl peptidase IV inhibitor | 2 | AY | [29-30],[692-693] |
| 8767 | dipeptidyl peptidase IV inhibitor (DPP IV inhibitor) | dipeptidyl peptidase IV inhibitor | 1 | DP | [236-237] |
| 8768 | dipeptidyl peptidase IV inhibitor (DPP IV inhibitor) | dipeptidyl peptidase IV inhibitor | 2 | DQ | [726-727],[751-752] |
| 8770 | dipeptidyl peptidase IV inhibitor (DPP IV inhibitor) | dipeptidyl peptidase IV inhibitor | 10 | EG | [136-137],[147-148],[153-154],[199-200],[319-320],[438-439],[465-466],[483-484],[506-507],[571-572] |
| 8772 | dipeptidyl peptidase IV inhibitor (DPP IV inhibitor) | dipeptidyl peptidase IV inhibitor | 1 | EI | [80-81] |
| 8775 | dipeptidyl peptidase IV inhibitor (DPP IV inhibitor) | dipeptidyl peptidase IV inhibitor | 1 | EV | [632-633] |
| 8777 | dipeptidyl peptidase IV inhibitor (DPP IV inhibitor) | dipeptidyl peptidase IV inhibitor | 1 | EY | [719-720] |
| 8781 | dipeptidyl peptidase IV inhibitor (DPP IV inhibitor) | dipeptidyl peptidase IV inhibitor | 15 | GE | [125-126],[146-147],[183-184],[191-192],[307-308],[318-319],[321-322],[437-438],[449-450],[464-465],[467-468],[482-483],[505-506],[511-512],[631-632] |
| 8782 | dipeptidyl peptidase IV inhibitor (DPP IV inhibitor) | dipeptidyl peptidase IV inhibitor | 7 | GF | [116-117],[163-164],[356-357],[365-366],[461-462],[605-606],[723-724] |
| 8783 | dipeptidyl peptidase IV inhibitor (DPP IV inhibitor) | dipeptidyl peptidase IV inhibitor | 24 | GG | [52-53],[96-97],[101-102],[115-116],[119-120],[225-226],[280-281],[298-299],[320-321],[347-348],[383-384],[387-388],[398-399],[423-424],[439-440],[446-447],[466-467],[484-485],[507-508],[525-526],[528-529],[574-575],[627-628],[630-631] |
| 8784 | dipeptidyl peptidase IV inhibitor (DPP IV inhibitor) | dipeptidyl peptidase IV inhibitor | 8 | GH | [157-158],[194-195],[238-239],[266-267],[350-351],[396-397],[440-441],[485-486] |
| 8785 | dipeptidyl peptidase IV inhibitor (DPP IV inhibitor) | dipeptidyl peptidase IV inhibitor | 6 | GI | [50-51],[174-175],[218-219],[258-259],[520-521],[687-688] |
| 8786 | dipeptidyl peptidase IV inhibitor (DPP IV inhibitor) | dipeptidyl peptidase IV inhibitor | 8 | GV | [97-98],[166-167],[188-189],[284-285],[550-551],[560-561],[612-613],[677-678] |
| 8788 | dipeptidyl peptidase IV inhibitor (DPP IV inhibitor) | dipeptidyl peptidase IV inhibitor | 2 | GY | [617-618],[768-769] |
| 8793 | dipeptidyl peptidase IV inhibitor (DPP IV inhibitor) | dipeptidyl peptidase IV inhibitor | 1 | HI | [694-695] |
| 8795 | dipeptidyl peptidase IV inhibitor (DPP IV inhibitor) | dipeptidyl peptidase IV inhibitor | 2 | HS | [195-196],[763-764] |
| 8798 | dipeptidyl peptidase IV inhibitor (DPP IV inhibitor) | dipeptidyl peptidase IV inhibitor | 1 | HW | [63-64] |
| 8802 | dipeptidyl peptidase IV inhibitor (DPP IV inhibitor) | dipeptidyl peptidase IV inhibitor | 1 | IL | [664-665] |
| 8808 | dipeptidyl peptidase IV inhibitor (DPP IV inhibitor) | dipeptidyl peptidase IV inhibitor | 4 | KE | [54-55],[67-68],[79-80],[570-571] |
| 8810 | dipeptidyl peptidase IV inhibitor (DPP IV inhibitor) | dipeptidyl peptidase IV inhibitor | 22 | KG | [95-96],[105-106],[240-241],[254-255],[257-258],[268-269],[349-350],[377-378],[380-381],[392-393],[395-396],[431-432],[442-443],[487-488],[504-505],[513-514],[519-520],[540-541],[554-555],[623-624],[698-699],[722-723] |
| 8812 | dipeptidyl peptidase IV inhibitor (DPP IV inhibitor) | dipeptidyl peptidase IV inhibitor | 1 | KI | [663-664] |
| 8813 | dipeptidyl peptidase IV inhibitor (DPP IV inhibitor) | dipeptidyl peptidase IV inhibitor | 2 | KK | [622-623],[721-722] |
| 8816 | dipeptidyl peptidase IV inhibitor (DPP IV inhibitor) | dipeptidyl peptidase IV inhibitor | 1 | KT | [614-615] |
| 8819 | dipeptidyl peptidase IV inhibitor (DPP IV inhibitor) | dipeptidyl peptidase IV inhibitor | 1 | KY | [625-626] |
| 8822 | dipeptidyl peptidase IV inhibitor (DPP IV inhibitor) | dipeptidyl peptidase IV inhibitor | 1 | LM | [706-707] |
| 8824 | dipeptidyl peptidase IV inhibitor (DPP IV inhibitor) | dipeptidyl peptidase IV inhibitor | 1 | LT | [456-457] |
| 8825 | dipeptidyl peptidase IV inhibitor (DPP IV inhibitor) | dipeptidyl peptidase IV inhibitor | 3 | LV | [13-14],[24-25],[647-648] |
| 8828 | dipeptidyl peptidase IV inhibitor (DPP IV inhibitor) | dipeptidyl peptidase IV inhibitor | 9 | MG | [49-50],[88-89],[182-183],[202-203],[382-383],[419-420],[597-598],[604-605],[634-635] |
| 8829 | dipeptidyl peptidase IV inhibitor (DPP IV inhibitor) | dipeptidyl peptidase IV inhibitor | 1 | MH | [86-87] |
| 8832 | dipeptidyl peptidase IV inhibitor (DPP IV inhibitor) | dipeptidyl peptidase IV inhibitor | 1 | ML | [403-404] |
| 8834 | dipeptidyl peptidase IV inhibitor (DPP IV inhibitor) | dipeptidyl peptidase IV inhibitor | 1 | MN | [290-291] |
| 8835 | dipeptidyl peptidase IV inhibitor (DPP IV inhibitor) | dipeptidyl peptidase IV inhibitor | 1 | MQ | [72-73] |
| 8836 | dipeptidyl peptidase IV inhibitor (DPP IV inhibitor) | dipeptidyl peptidase IV inhibitor | 1 | MR | [707-708] |
| 8837 | dipeptidyl peptidase IV inhibitor (DPP IV inhibitor) | dipeptidyl peptidase IV inhibitor | 1 | MV | [1-2] |
| 8838 | dipeptidyl peptidase IV inhibitor (DPP IV inhibitor) | dipeptidyl peptidase IV inhibitor | 1 | MY | [714-715] |
| 8840 | dipeptidyl peptidase IV inhibitor (DPP IV inhibitor) | dipeptidyl peptidase IV inhibitor | 1 | ND | [709-710] |
| 8843 | dipeptidyl peptidase IV inhibitor (DPP IV inhibitor) | dipeptidyl peptidase IV inhibitor | 7 | NG | [100-101],[291-292],[337-338],[460-461],[629-630],[637-638],[667-668] |
| 8851 | dipeptidyl peptidase IV inhibitor (DPP IV inhibitor) | dipeptidyl peptidase IV inhibitor | 1 | NV | [701-702] |
| 8853 | dipeptidyl peptidase IV inhibitor (DPP IV inhibitor) | dipeptidyl peptidase IV inhibitor | 1 | NY | [671-672] |
| 8854 | dipeptidyl peptidase IV inhibitor (DPP IV inhibitor) | dipeptidyl peptidase IV inhibitor | 2 | PF | [5-6],[651-652] |
| 8855 | dipeptidyl peptidase IV inhibitor (DPP IV inhibitor) | dipeptidyl peptidase IV inhibitor | 78 | PG | [65-66],[118-119],[133-134],[142-143],[150-151],[156-157],[162-163],[165-166],[170-171],[173-174],[176-177],[179-180],[187-188],[190-191],[211-212],[214-215],[217-218],[228-229],[231-232],[237-238],[243-244],[251-252],[262-263],[265-266],[274-275],[277-278],[283-284],[288-289],[297-298],[300-301],[303-304],[306-307],[323-324],[326-327],[329-330],[334-335],[340-341],[343-344],[346-347],[352-353],[355-356],[358-359],[361-362],[364-365],[369-370],[374-375],[386-387],[389-390],[401-402],[407-408],[422-423],[428-429],[445-446],[451-452],[463-464],[469-470],[490-491],[493-494],[498-499],[501-502],[510-511],[522-523],[534-535],[537-538],[546-547],[549-550],[559-560],[562-563],[573-574],[577-578],[580-581],[583-584],[586-587],[589-590],[592-593],[608-609],[686-687],[738-739] |
| 8856 | dipeptidyl peptidase IV inhibitor (DPP IV inhibitor) | dipeptidyl peptidase IV inhibitor | 3 | PH | [44-45],[70-71],[93-94] |
| 8857 | dipeptidyl peptidase IV inhibitor (DPP IV inhibitor) | dipeptidyl peptidase IV inhibitor | 2 | PI | [453-454],[530-531] |
| 8858 | dipeptidyl peptidase IV inhibitor (DPP IV inhibitor) | dipeptidyl peptidase IV inhibitor | 6 | PK | [253-254],[376-377],[391-392],[430-431],[503-504],[518-519] |
| 8859 | dipeptidyl peptidase IV inhibitor (DPP IV inhibitor) | dipeptidyl peptidase IV inhibitor | 3 | PM | [201-202],[418-419],[773-774] |
| 8860 | dipeptidyl peptidase IV inhibitor (DPP IV inhibitor) | dipeptidyl peptidase IV inhibitor | 1 | PN | [636-637] |
| 8861 | dipeptidyl peptidase IV inhibitor (DPP IV inhibitor) | dipeptidyl peptidase IV inhibitor | 8 | PQ | [37-38],[58-59],[75-76],[82-83],[207-208],[480-481],[515-516],[674-675] |
| 8862 | dipeptidyl peptidase IV inhibitor (DPP IV inhibitor) | dipeptidyl peptidase IV inhibitor | 3 | PS | [110-111],[138-139],[749-750] |
| 8863 | dipeptidyl peptidase IV inhibitor (DPP IV inhibitor) | dipeptidyl peptidase IV inhibitor | 4 | PT | [42-43],[474-475],[567-568],[594-595] |
| 8864 | dipeptidyl peptidase IV inhibitor (DPP IV inhibitor) | dipeptidyl peptidase IV inhibitor | 3 | PV | [477-478],[654-655],[712-713] |
| 8867 | dipeptidyl peptidase IV inhibitor (DPP IV inhibitor) | dipeptidyl peptidase IV inhibitor | 2 | QA | [727-728],[752-753] |
| 8868 | dipeptidyl peptidase IV inhibitor (DPP IV inhibitor) | dipeptidyl peptidase IV inhibitor | 1 | QD | [103-104] |
| 8871 | dipeptidyl peptidase IV inhibitor (DPP IV inhibitor) | dipeptidyl peptidase IV inhibitor | 9 | QG | [127-128],[205-206],[208-209],[279-280],[311-312],[331-332],[448-449],[481-482],[516-517] |
| 8872 | dipeptidyl peptidase IV inhibitor (DPP IV inhibitor) | dipeptidyl peptidase IV inhibitor | 3 | QH | [35-36],[39-40],[56-57] |
| 8874 | dipeptidyl peptidase IV inhibitor (DPP IV inhibitor) | dipeptidyl peptidase IV inhibitor | 2 | QL | [18-19],[747-748] |
| 8875 | dipeptidyl peptidase IV inhibitor (DPP IV inhibitor) | dipeptidyl peptidase IV inhibitor | 3 | QN | [336-337],[459-460],[670-671] |
| 8876 | dipeptidyl peptidase IV inhibitor (DPP IV inhibitor) | dipeptidyl peptidase IV inhibitor | 3 | QQ | [38-39],[59-60],[204-205] |
| 8878 | dipeptidyl peptidase IV inhibitor (DPP IV inhibitor) | dipeptidyl peptidase IV inhibitor | 2 | QT | [107-108],[675-676] |
| 8881 | dipeptidyl peptidase IV inhibitor (DPP IV inhibitor) | dipeptidyl peptidase IV inhibitor | 3 | QY | [73-74],[76-77],[760-761] |
| 8882 | dipeptidyl peptidase IV inhibitor (DPP IV inhibitor) | dipeptidyl peptidase IV inhibitor | 2 | RG | [234-235],[472-473] |
| 8883 | dipeptidyl peptidase IV inhibitor (DPP IV inhibitor) | dipeptidyl peptidase IV inhibitor | 1 | RH | [32-33] |
| 8885 | dipeptidyl peptidase IV inhibitor (DPP IV inhibitor) | dipeptidyl peptidase IV inhibitor | 1 | RK | [78-79] |
| 8888 | dipeptidyl peptidase IV inhibitor (DPP IV inhibitor) | dipeptidyl peptidase IV inhibitor | 1 | RN | [708-709] |
| 8891 | dipeptidyl peptidase IV inhibitor (DPP IV inhibitor) | dipeptidyl peptidase IV inhibitor | 1 | SF | [765-766] |
| 8892 | dipeptidyl peptidase IV inhibitor (DPP IV inhibitor) | dipeptidyl peptidase IV inhibitor | 1 | SH | [47-48] |
| 8893 | dipeptidyl peptidase IV inhibitor (DPP IV inhibitor) | dipeptidyl peptidase IV inhibitor | 1 | SI | [409-410] |
| 8898 | dipeptidyl peptidase IV inhibitor (DPP IV inhibitor) | dipeptidyl peptidase IV inhibitor | 1 | TD | [595-596] |
| 8901 | dipeptidyl peptidase IV inhibitor (DPP IV inhibitor) | dipeptidyl peptidase IV inhibitor | 4 | TG | [457-458],[475-476],[568-569],[676-677] |
| 8903 | dipeptidyl peptidase IV inhibitor (DPP IV inhibitor) | dipeptidyl peptidase IV inhibitor | 1 | TI | [108-109] |
| 8904 | dipeptidyl peptidase IV inhibitor (DPP IV inhibitor) | dipeptidyl peptidase IV inhibitor | 1 | TK | [539-540] |
| 8911 | dipeptidyl peptidase IV inhibitor (DPP IV inhibitor) | dipeptidyl peptidase IV inhibitor | 1 | TT | [649-650] |
| 8912 | dipeptidyl peptidase IV inhibitor (DPP IV inhibitor) | dipeptidyl peptidase IV inhibitor | 1 | TV | [741-742] |
| 8914 | dipeptidyl peptidase IV inhibitor (DPP IV inhibitor) | dipeptidyl peptidase IV inhibitor | 1 | TY | [716-717] |
| 8915 | dipeptidyl peptidase IV inhibitor (DPP IV inhibitor) | dipeptidyl peptidase IV inhibitor | 1 | VD | [551-552] |
| 8917 | dipeptidyl peptidase IV inhibitor (DPP IV inhibitor) | dipeptidyl peptidase IV inhibitor | 2 | VF | [660-661],[678-679] |
| 8918 | dipeptidyl peptidase IV inhibitor (DPP IV inhibitor) | dipeptidyl peptidase IV inhibitor | 5 | VG | [114-115],[167-168],[285-286],[478-479],[655-656] |
| 8919 | dipeptidyl peptidase IV inhibitor (DPP IV inhibitor) | dipeptidyl peptidase IV inhibitor | 2 | VH | [25-26],[762-763] |
| 8921 | dipeptidyl peptidase IV inhibitor (DPP IV inhibitor) | dipeptidyl peptidase IV inhibitor | 1 | VK | [613-614] |
| 8922 | dipeptidyl peptidase IV inhibitor (DPP IV inhibitor) | dipeptidyl peptidase IV inhibitor | 4 | VL | [15-16],[21-22],[600-601],[733-734] |
| 8923 | dipeptidyl peptidase IV inhibitor (DPP IV inhibitor) | dipeptidyl peptidase IV inhibitor | 2 | VM | [633-634],[713-714] |
| 8924 | dipeptidyl peptidase IV inhibitor (DPP IV inhibitor) | dipeptidyl peptidase IV inhibitor | 1 | VN | [99-100] |
| 8927 | dipeptidyl peptidase IV inhibitor (DPP IV inhibitor) | dipeptidyl peptidase IV inhibitor | 1 | VT | [648-649] |
| 8928 | dipeptidyl peptidase IV inhibitor (DPP IV inhibitor) | dipeptidyl peptidase IV inhibitor | 1 | VW | [702-703] |
| 8932 | dipeptidyl peptidase IV inhibitor (DPP IV inhibitor) | dipeptidyl peptidase IV inhibitor | 1 | YA | [757-758] |
| 8933 | dipeptidyl peptidase IV inhibitor (DPP IV inhibitor) | dipeptidyl peptidase IV inhibitor | 1 | YD | [717-718] |
| 8935 | dipeptidyl peptidase IV inhibitor (DPP IV inhibitor) | dipeptidyl peptidase IV inhibitor | 1 | YF | [690-691] |
| 8936 | dipeptidyl peptidase IV inhibitor (DPP IV inhibitor) | dipeptidyl peptidase IV inhibitor | 1 | YG | [626-627] |
| 8937 | dipeptidyl peptidase IV inhibitor (DPP IV inhibitor) | dipeptidyl peptidase IV inhibitor | 1 | YH | [693-694] |
| 8939 | dipeptidyl peptidase IV inhibitor (DPP IV inhibitor) | dipeptidyl peptidase IV inhibitor | 1 | YK | [720-721] |
| 8940 | dipeptidyl peptidase IV inhibitor (DPP IV inhibitor) | dipeptidyl peptidase IV inhibitor | 2 | YL | [745-746],[769-770] |
| 8942 | dipeptidyl peptidase IV inhibitor (DPP IV inhibitor) | dipeptidyl peptidase IV inhibitor | 2 | YN | [666-667],[672-673] |
| 8944 | dipeptidyl peptidase IV inhibitor (DPP IV inhibitor) | dipeptidyl peptidase IV inhibitor | 2 | YR | [31-32],[77-78] |
| 8946 | dipeptidyl peptidase IV inhibitor (DPP IV inhibitor) | dipeptidyl peptidase IV inhibitor | 1 | YV | [761-762] |
| 8948 | dipeptidyl peptidase IV inhibitor (DPP IV inhibitor) | dipeptidyl peptidase IV inhibitor | 2 | YY | [30-31],[689-690] |
| 9116 | dipeptidyl peptidase IV inhibitor (DPP IV inhibitor) | dipeptidyl peptidase IV inhibitor | 1 | GPV | [476-478] |
| 9117 | dipeptidyl peptidase IV inhibitor (DPP IV inhibitor) | dipeptidyl peptidase IV inhibitor | 2 | GPM | [200-202],[417-419] |
| 9338 | Dipeptidyl peptidase IV inhibitor | dipeptidyl peptidase IV inhibitor | 4 | GPAG | [128-131],[143-146],[315-318],[411-414] |

**PeptideRanker Results of 118 ACE inhibitor peptides**

PeptideRanker orders the peptides entered according to their score

|  | [**Sequence**](http://bioware.ucd.ie/~compass/biowareweb/cgi-bin/PHP_helper_files/peptiderankerInfo.php?jobId=dL8ah0) | [**Score**](http://bioware.ucd.ie/~compass/biowareweb/cgi-bin/PHP_helper_files/peptiderankerInfo.php?jobId=dL8ah0) |
| --- | --- | --- |
| 0 | GF | 0.99 |
| 1 | FP | 0.99 |
| 2 | FG | 0.99 |
| 3 | AF | 0.97 |
| 4 | GPM | 0.96 |
| 5 | SF | 0.95 |
| 6 | GM | 0.95 |
| 7 | MGP | 0.94 |
| 8 | MG | 0.94 |
| 9 | GPP | 0.93 |
| 10 | GP | 0.91 |
| 11 | PP | 0.89 |
| 12 | GPL | 0.89 |
| 13 | GG | 0.89 |
| 14 | PG | 0.88 |
| 15 | PLP | 0.86 |
| 16 | PGL | 0.86 |
| 17 | GLP | 0.86 |
| 18 | FGK | 0.85 |
| 19 | PLPLL | 0.84 |
| 20 | MY | 0.84 |
| 21 | VF | 0.82 |
| 22 | PL | 0.81 |
| 23 | GL | 0.81 |
| 24 | VW | 0.80 |
| 25 | PLG | 0.80 |
| 26 | LPG | 0.80 |
| 27 | PR | 0.79 |
| 28 | LGP | 0.79 |
| 29 | GR | 0.77 |
| 30 | YGG | 0.76 |
| 31 | YP | 0.74 |
| 32 | RG | 0.74 |
| 33 | GY | 0.74 |
| 34 | GPA | 0.73 |
| 35 | AGP | 0.73 |
| 36 | LG | 0.72 |
| 37 | GLY | 0.71 |
| 38 | YG | 0.69 |
| 39 | SGP | 0.68 |
| 40 | LYP | 0.68 |
| 41 | QGP | 0.67 |
| 42 | GQP | 0.64 |
| 43 | GHG | 0.64 |
| 44 | AP | 0.63 |
| 45 | DM | 0.61 |
| 46 | FDK | 0.59 |
| 47 | YL | 0.58 |
| 48 | IP | 0.58 |
| 49 | HP | 0.57 |
| 50 | GKP | 0.57 |
| 51 | AG | 0.55 |
| 52 | PH | 0.54 |
| 53 | KGP | 0.53 |
| 54 | GH | 0.53 |
| 55 | LY | 0.52 |
| 56 | GI | 0.52 |
| 57 | GA | 0.52 |
| 58 | VPP | 0.51 |
| 59 | IG | 0.50 |
| 60 | HG | 0.49 |
| 61 | GPV | 0.47 |
| 62 | TGP | 0.46 |
| 63 | VGP | 0.41 |
| 64 | SG | 0.41 |
| 65 | LQP | 0.41 |
| 66 | QG | 0.39 |
| 67 | PQ | 0.39 |
| 68 | NG | 0.39 |
| 69 | KP | 0.39 |
| 70 | IL | 0.39 |
| 71 | GD | 0.39 |
| 72 | DG | 0.39 |
| 73 | GQ | 0.38 |
| 74 | HL | 0.37 |
| 75 | AY | 0.35 |
| 76 | GS | 0.34 |
| 77 | VLP | 0.33 |
| 78 | GEP | 0.33 |
| 79 | IY | 0.32 |
| 80 | LA | 0.31 |
| 81 | KG | 0.31 |
| 82 | VVF | 0.30 |
| 83 | GK | 0.30 |
| 84 | YH | 0.29 |
| 85 | TP | 0.27 |
| 86 | YA | 0.26 |
| 87 | PT | 0.25 |
| 88 | YN | 0.24 |
| 89 | VVPP | 0.24 |
| 90 | VP | 0.24 |
| 91 | KL | 0.23 |
| 92 | GT | 0.23 |
| 93 | NY | 0.22 |
| 94 | EQGP | 0.22 |
| 95 | LQ | 0.20 |
| 96 | TG | 0.19 |
| 97 | AA | 0.19 |
| 98 | KY | 0.18 |
| 99 | GV | 0.18 |
| 100 | VG | 0.17 |
| 101 | EKGP | 0.16 |
| 102 | YK | 0.15 |
| 103 | AVL | 0.14 |
| 104 | GE | 0.11 |
| 105 | KA | 0.10 |
| 106 | HK | 0.10 |
| 107 | EG | 0.10 |
| 108 | YV | 0.09 |
| 109 | EY | 0.07 |
| 110 | AV | 0.06 |
| 111 | EI | 0.05 |
| 112 | EA | 0.04 |
| 113 | AVV | 0.04 |
| 114 | VK | 0.03 |
| 115 | KE | 0.03 |
| 116 | EV | 0.02 |
| 117 | EK | 0.02 |

**All possible enzymatic cleavages of salmon col8a1 by pepsin and trypsin**

**--ExPASy PeptideCutter Result**


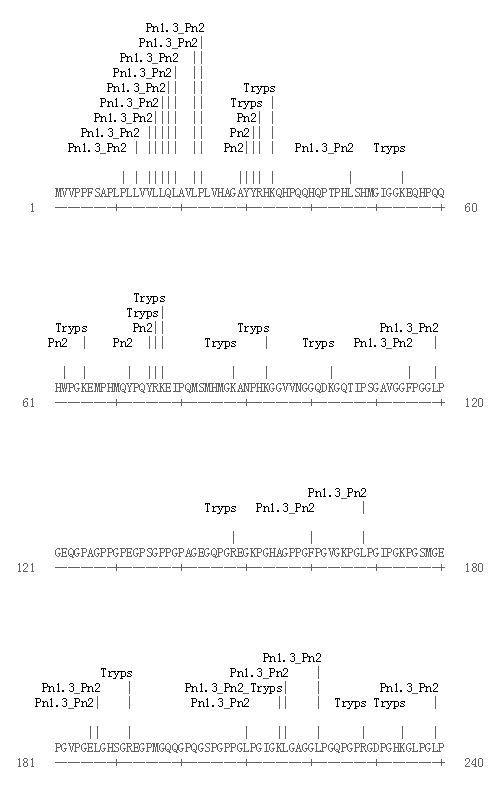

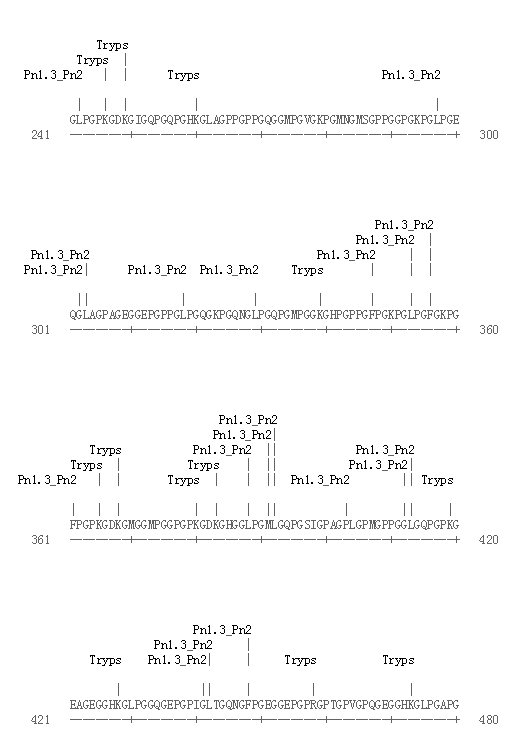

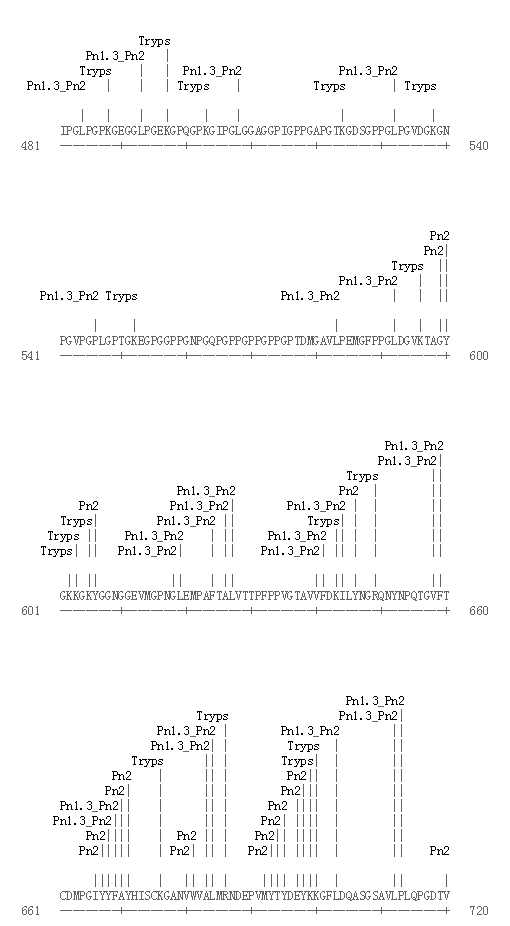

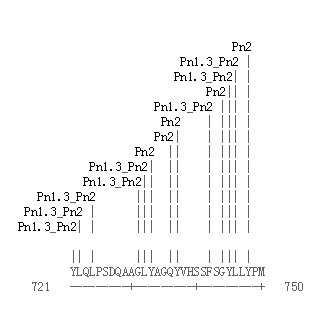

Supplement: Supplementary file 1 [file marinedrugs-16-00365-s001.zip › Supplementary_revised/Table S10.docx]
