# Supplementary material for: High Throughput Identification of Antihypertensive Peptides from Fish Proteome Datasets
Source: Mar Drugs. 2018 Oct 2;16(10):365. doi: 10.3390/md16100365 (PMC6212880; doi:10.3390/md16100365)
Supplement: Supplementary file 1 [file marinedrugs-16-00365-s001.zip › Supplementary_revised/Table S2.docx]

Table S2. Websites for downloading the protein datasets of 18 fish species.

| Species | Common name | Websites |
| --- | --- | --- |
| *Astyanax mexicanus* | Mexican tetra | ftp://ftp.ensembl.org/pub/release-91/fasta/astyanax_mexicanus/pep/ |
| *Boleophthalmus pectinirostris* | BP, Blue-spotted mudskipper | ftp://ftp.ncbi.nlm.nih.gov/genomes/all/GCF/000/788/275/GCF_000788275.1_BP.fa/ |
| *Ctenopharyngodon idella* | Grass carp | http://www.ncgr.ac.cn/grasscarp/ |
| *Danio rerio* | Zebrafish | ftp://ftp.ensembl.org/pub/release-91/fasta/danio_rerio/pep/ |
| *Gadus morhua* | Atlantic cod | ftp://ftp.ensembl.org/pub/release-91/fasta/gadus_morhua/pep/ |
| *Gasterosteus aculeatus* | Three-spined stickleback | ftp://ftp.ensembl.org/pub/release-91/fasta/gasterosteus_aculeatus/pep/ |
| *Latimeria chalumnae* | Coelacanth | ftp://ftp.ncbi.nlm.nih.gov/genomes/all/GCF/000/225/785/GCF_000225785.1_LatCha1/ |
| *Lepisosteus oculatus* | Spotted gar | ftp://ftp.ensembl.org/pub/release-91/fasta/lepisosteus_oculatus/pep/ |
| *Oreochromis niloticus* | Nile tilapia | ftp://ftp.ensembl.org/pub/release-91/fasta/oreochromis_niloticus/pep/ |
| *Oryzias latipes* | Medaka | ftp://ftp.ensembl.org/pub/release-91/fasta/oryzias_latipes/pep/ |
| *Periophthalmus magnuspinnatus* | PM, Giant-fin mudskipper | ftp://ftp.ncbi.nlm.nih.gov/genomes/all/GCA/000/787/105/GCA_000787105.1_PM.fa/ |
| *Salmo salar* | Atlantic salmon | ftp://ftp.ncbi.nlm.nih.gov/genomes/all/GCF/000/233/375/GCF_000233375.1_ICSASG_v2 |
| *Sinocyclocheilus anshuiensis* | Sa, Golden-line barbel | ftp://ftp.ncbi.nlm.nih.gov/genomes/all/GCF/001/515/605/GCF_001515605.1_SAMN03320099.WGS_v1.1 |
| *Sinocyclocheilus grahami* | Sg, Golden-line barbel | ftp://ftp.ncbi.nlm.nih.gov/genomes/all/GCF/001/515/645/GCF_001515645.1_SAMN03320097.WGS_v1.1 |
| *Sinocyclocheilus rhinocerous* | Sr, Golden-line barbel | ftp://ftp.ncbi.nlm.nih.gov/genomes/all/GCF/001/515/625/GCF_001515625.1_SAMN03320098_v1.1 |
| *Takifugu rubripes* | Japanese pufferfish | ftp://ftp.ensembl.org/pub/release-91/fasta/takifugu_rubripes/pep/ |
| *Tetraodon nigroviridis* | Green spotted puffer | ftp://ftp.ensembl.org/pub/release-91/fasta/tetraodon_nigroviridis/pep/ |
| *Xiphophorus maculatus* | Southern platyfish, moonfish | ftp://ftp.ensembl.org/pub/release-91/fasta/xiphophorus_maculatus/pep/ |
