# Supplementary material for: High Throughput Identification of Antihypertensive Peptides from Fish Proteome Datasets
Source: Mar Drugs. 2018 Oct 2;16(10):365. doi: 10.3390/md16100365 (PMC6212880; doi:10.3390/md16100365)
Supplement: Supplementary file 1 [file marinedrugs-16-00365-s001.zip › Supplementary_revised/Table S7.docx]

Table S7. Accession numbers of collagen subunit protein sequences and water ecotypes of 33 fish species

| Species | Common name | Accession No. of Col4a5 | Accession No. of Col8a1 | Water ecotypes |
| --- | --- | --- | --- | --- |
| *Lepisosteus oculatus^a^* | Spotted gar | XP_015206871.1 | ENSLOCP00000011536.1 | Freshwater; brackish |
| *Danio rerio^a^* | Zebrafish | XP_005166559.1 | XP_690914.3 | Freshwater |
| *Gasterosteus aculeatus^a^* | Three-spined stickleback |  | ENSGACP00000003642.1 | Marine; freshwater; brackish |
| *Oryzias latipes^a^* | Japanese rice fish, Medaka | XP_011485964.1 | XP_004081516.1 | Freshwater; brackish |
| *Takifugu rubripes^a^* | Japanese pufferfish | XP_011604935.1 | XP_003961858.1 | Marine; freshwater; brackish |
| *Ctenopharyngodon idella^a^* | Grass carp |  | CI01000015_06858948_06861652 | Freshwater |
| *Scleropages formosus* | Asian arowana |  | XP_018605480.1 | Freshwater |
| *Larimichthys crocea* | Large yellow croaker | XP_019116036.1 | XP_010751166.2 | marine |
| *Oryzias melastigma* | Marine medaka | XP_024143783.1 | XP_024142958.1 | Freshwater; brackish |
| *Oreochromis niloticus^a^* | Nile tilapia | XP_005464118.1 | XP_005475554.1 | Freshwater; brackish |
| *Cynoglossus semilaevis* | Tongue sole | XP_024918491.1 | XP_008326417.1 | Marine; freshwater; brackish |
| *Gadus morhua^a^* | Atlantic cod |  | ENSGMOP00000017893.1 | Marine; brackish |
| *Clupea harengus* | Atlantic herring |  | XP_012692753.1 | Marine; brackish |
| *Salmo salar^a^* | Atlantic salmon | XP_014062615.1 | XP_014029720.1 | Marine; freshwater; brackish |
| *Oncorhynchus kisutch* | Coho salmon |  | XP_020355464.1 | Marine; freshwater; brackish |
| *Sinocyclocheilus grahami^a^* | Sg, Golden-line barbel | XP_016096442.1 | XP_016113463.1 | Freshwater |
| *Sinocyclocheilus anshuiensis^a^* | Sa, Golden-line barbel | XP_016336831.1 | XP_016315123.1 | Freshwater |
| *Sinocyclocheilus rhinocerous^a^* | Sr, Golden-line barbel | XP_016372043.1 | XP_016383547.1 | Freshwater |
| *Ictalurus punctatus* | Channel catfish | XP_017326951.1 |  | Freshwater |
| *Astyanax mexicanus^a^* | Mexican tetra | XP_022534175.1 |  | Freshwater |
| *Hippocampus comes* | Tiger tail seahorse | XP_019711649.1 | XP_019749992.1 | Marine |
| *Paralichthys olivaceus* | Bastard halibut, Olive flounder | XP_019961846.1 | XP_019956235.1 | Marine |
| *Oncorhynchus mykiss* | Rainbow trout | XP_021433329.1 |  | Marine; freshwater; brackish |
| *Monopterus albus* | Asian swamp eel | XP_020479362.1 | XP_020465181.1 | Freshwater; brackish |
| *Labrus bergylta* | Ballan wrasse |  | XP_020506896.1 | Marine |
| *Acanthochromis polyacanthus* | Spiny chromis damselfish | XP_022057858.1 | XP_022077904.1 | Marine |
| *Stegastes partitus* | Bicolor damselfish |  | XP_008299579.1 | marine |
| *Xiphophorus maculatus^a^* | Southern platyfish, moonfish | XP_023201800.1 | XP_023192266.1 | Freshwater |
| *Fundulus heteroclitus* | Mummichog | XP_012730418.1 | XP_021173149.1 | Marine; freshwater; brackish |
| *Periophthalmus magnuspinnatus^a^* | PM, Giant-fin mudskipper |  | Pm_GLEAN_10021965^b^ | Marine; freshwater; brackish |
| *Boleophthalmus pectinirostris^a^* | BP, Blue-spotted mudskipper | XP_020778420.1 | XP_020791982.1 | Marine; freshwater; brackish |
| *Seriola dumerili* | Greater amberjack | XP_022617014.1 |  | Marine |
| *Lates calcarifer* | Barramundi | XP_018559310.1 |  | Marine; freshwater; brackish |

**^a^** Species employed in our previous *in situ* mapping analysis. **^b^** Sequence identified in our local protein dataset.
